# TP53 mutations and protein immunopositivity may predict for poor outcome but also for trastuzumab benefit in patients with early breast cancer treated in the adjuvant setting

**DOI:** 10.18632/oncotarget.9022

**Published:** 2016-04-26

**Authors:** George Fountzilas, Eleni Giannoulatou, Zoi Alexopoulou, Flora Zagouri, Eleni Timotheadou, Kyriaki Papadopoulou, Sotiris Lakis, Mattheos Bobos, Christos Poulios, Maria Sotiropoulou, Aggeliki Lyberopoulou, Helen Gogas, George Pentheroudakis, Dimitrios Pectasides, Angelos Koutras, Christos Christodoulou, Christos Papandreou, Epaminontas Samantas, Pavlos Papakostas, Paris Kosmidis, Dimitrios Bafaloukos, Charisios Karanikiotis, Meletios-Athanassios Dimopoulos, Vassiliki Kotoula

**Affiliations:** ^1^ Laboratory of Molecular Oncology, Hellenic Foundation for Cancer Research/Aristotle University of Thessaloniki, Thessaloniki, Greece; ^2^ Aristotle University of Thessaloniki, Thessaloniki, Greece; ^3^ Victor Chang Cardiac Research Institute, Darlinghurst, NSW, Australia; ^4^ The University of New South Wales, NSW, Australia; ^5^ Department of Biostatistics, Health Data Specialists Ltd, Athens, Greece; ^6^ Department of Clinical Therapeutics, “Alexandra” Hospital, National and Kapodistrian University of Athens School of Medicine, Athens, Greece; ^7^ Department of Medical Oncology, “Papageorgiou” Hospital, Aristotle University of Thessaloniki, School of Health Sciences, Faculty of Medicine, Thessaloniki, Greece; ^8^ Department of Pathology, Aristotle University of Thessaloniki, School of Health Sciences, Faculty of Medicine, Thessaloniki, Greece; ^9^ Department of Pathology, “Alexandra” Hospital, Athens, Greece; ^10^ First Department of Medicine, “Laiko” General Hospital, National and Kapodistrian University of Athens School of Medicine, Athens, Greece; ^11^ Department of Medical Oncology, Ioannina University Hospital, Ioannina, Greece; ^12^ Oncology Section, Second Department of Internal Medicine, “Hippokration” Hospital, Athens, Greece; ^13^ Division of Oncology, Department of Medicine, University Hospital, University of Patras Medical School, Patras, Greece; ^14^ Second Department of Medical Oncology, “Metropolitan” Hospital, Piraeus, Greece; ^15^ Department of Medical Oncology, University Hospital of Larissa, University of Thessaly School of Medicine, Larissa, Greece; ^16^ Third Department of Medical Oncology, “Agii Anargiri” Cancer Hospital, Athens, Greece; ^17^ Oncology Unit, “Hippokration” Hospital, Athens, Greece; ^18^ Second Department of Medical Oncology, Hygeia Hospital, Athens, Greece; ^19^ First Department of Medical Oncology, “Metropolitan” Hospital, Piraeus, Greece; ^20^ Department of Medical Oncology, 424 Army General Hospital, Thessaloniki, Greece

**Keywords:** early breast cancer, TP53 mutations, PIK3CA mutations, p53 immunohistochemistry, trastuzumab

## Abstract

**Background:**

We investigated the impact of PIK3CA and TP53 mutations and p53 protein status on the outcome of patients who had been treated with adjuvant anthracycline-taxane chemotherapy within clinical trials in the pre- and post-trastuzumab era.

**Results:**

TP53 and PIK3CA mutations were found in 380 (21.5%) and 458 (25.9%) cases, respectively, including 104 (5.9%) co-mutated tumors; p53 immunopositivity was observed in 848 tumors (53.5%). TP53 mutations (*p* < 0.001) and p53 protein positivity (*p* = 0.001) were more frequent in HER2-positive and triple negative (TNBC) tumors, while PIK3CA mutations were more frequent in Luminal A/B tumors (*p* < 0.001). TP53 mutation status and p53 protein expression but not PIK3CA mutation status interacted with trastuzumab treatment for disease-free survival; patients with tumors bearing TP53 mutations or immunopositive for p53 protein fared better when treated with trastuzumab, while among patients treated with trastuzumab those with the above characteristics fared best (interaction *p* = 0.017 for mutations; *p* = 0.015 for IHC). Upon multivariate analysis the above interactions remained significant in HER2-positive patients; in the entire cohort, TP53 mutations were unfavorable in patients with Luminal A/B (*p* = 0.003) and TNBC (*p* = 0.025); p53 immunopositivity was strongly favorable in patients treated with trastuzumab (*p* = 0.009).

**Materials and Methods:**

TP53 and PIK3CA mutation status was examined in 1766 paraffin tumor DNA samples with informative semiconductor sequencing results. Among these, 1585 cases were also informative for p53 protein status assessed by immunohistochemistry (IHC; 10% positivity cut-off).

**Conclusions:**

TP53 mutations confer unfavorable prognosis in patients with Luminal A/B and TNBC tumors, while p53 immunopositivity may predict for trastuzumab benefit in the adjuvant setting.

## INTRODUCTION

Breast cancer is the most common malignancy and the leading cause of death from cancer among women worldwide [1]. It is estimated that during 2015 approximately 232,000 new cases will be diagnosed and 40,000 deaths will occur in the US [2].

In almost all randomized trials that evaluated different agents or combinations in the adjuvant setting in the past decades, breast cancer was treated as a single clinical entity in terms of chemotherapy; tumor biological characteristics that were addressed for additional targeted drugs were positivity for hormone receptors (ER/PgR) and later on, HER2 protein overexpression / gene amplification. Eventually, however, following initial molecular subtyping [3–5], researchers realized that breast cancer is a heterogeneous group of diseases, displaying distinct biology, responses to various treatments and clinical outcomes [6]. Recently, whole genome and exome sequencing studies have contributed to a wealth of genomic and comprehensive molecular alteration data for almost every type of cancer, including breast cancer [7], providing an unprecedented view to the inter- and intra-tumoral heterogeneity of these malignancies [8]. Ultimately, the major incentive for investigating the impact of (somatic) mutations in tumor behavior is the potential for the development of effective targeted anticancer treatments. Unfortunately, the technology needed for the above impressive achievements is not easily applicable in large trials, since it is costly and requires fresh-frozen tissue, which is mostly not available. To overcome these hurdles, investigators have been increasingly adapting methods and markers for application on formalin-fixed paraffin-embedded (FFPE) tumor tissues [9].

As revealed in whole genome and exome studies, PIK3CA and TP53 are by far the most commonly mutated genes in breast cancer [10, 11]. The tumor suppressor TP53 gene [12], located on the short arm of chromosome 17 (17p13.1), is mutated in approximately 30% of breast cancers [13]. Even though over 4,300 different TP53 mutations have been reported in patients with cancer, novel mutations are continuously being identified [14], while mutation rates between intrinsic or immunophenotypical tumor subtypes vary [10, 15]. Most somatic TP53 mutations are located in the region coding for the DNA binding domain (DBD) of the protein [16]. TP53 mutations are associated with bad prognosis in breast cancer, whereby the position and type of mutation may be important for patient outcome (reviewed in [17, 18]). TP53 mutations are characterized as loss-of-function or gain-of-function but the result of practically all TP53 mutations is stabilization of the mutant protein [19]. The short-lived wild-type p53 protein [20] is unnoticed in surgical specimens with immunohistochemistry, but the stabilized mutant protein accumulates and can be demonstrated, provided that the detector antibody can bind to the mutant epitope. Of note, nuclear p53 protein accumulation, which only partially reflects TP53 mutation status, as shown in earlier studies [21, 22], was also reported to adversely impact breast cancer patient outcome [23–25].

The phosphatidylinositol-4,5-bisphosphonate 3-kinase catalytic subunit alpha (PIK3CA) gene, located in the long arm of chromosome 3 (3q26.32), is mutated in approximately 27% of breast carcinomas [26]. PIK3CA mutations are reported to cluster within the helical domain (exon 10; coding exon 9) and the kinase domain (exon 21, coding exon 20) [27]. Importantly, PIK3CA mutations have been associated with favorable clinicopathological parameters, i.e. ER expression, smaller tumor size and low histological grade, as well as good prognosis [28, 29].

Even though the prognostic/predictive role of TP53 and PIK3CA mutations has been addressed in a considerable number of studies, information regarding their role with respect to specific breast cancer subtypes within the context of adjuvant trials remains limited. Our Group has recently initiated a large study evaluating the impact of tumor mutation patterns on breast cancer patient outcome with respect to clinical subtypes and treatment. FFPE tumor DNA from patients participating in four adjuvant trials conducted by our Group, was sequenced for a panel of gene targets based on the incidence of previously identified recurrently mutated genes in breast cancer [10, 11, 30]. Herein, we present our findings on TP53 and PIK3CA mutations. In addition, because of the above cited possible unfavorable prognostic effect of p53 protein immunopositivity in good prognosis breast cancer [23, 24], we compared this marker with TP53 mutations, as well.

## RESULTS

### Distribution of TP53 and PIK3CA mutations in tumor tissues

**Figure 1:**
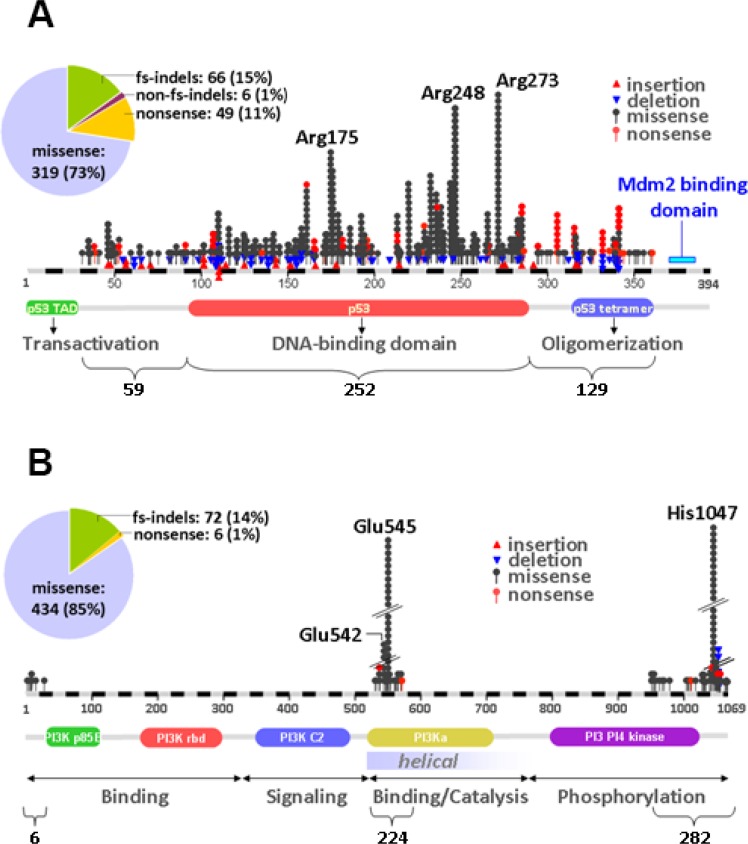
Distribution of TP53 and PIK3CA mutations in early breast cancer (**A**) TP53 mutations were dispersed throughout the coding region but aggregated in the area coding for the DNA binding domain of the protein. (**B**) PIK3CA mutations were more common in the kinase domain. In the pies in A and B, the distribution of mutation types per gene is shown. fs: frameshift; indels: insertions/deletions.

Mutations were considered for amino acid changing variants in coding regions with minor allele frequency (MAF) < 0.1% in the case of registered single nucleotide polymorphisms (SNPs). For PIK3CA, 512 amino acid changing variants were mutations and for TP53 440. The majority of mutations were missense for both genes; frameshift indels occurred in PIK3CA as well, while nonsense mutations were significantly more frequent in TP53 (49/440) than in PIK3CA (6/512), in line with the tumor suppressor and oncogenic nature of these genes, respectively (Figure 1). TP53 mutations were mostly found in the coding region corresponding to the DNA binding domain (DBD) of the protein, followed by mutations in the oligomerization and the the transactivation (TAD) domains; mutations were present almost throughout the TP53 coding region except for the MDM2-binding domain, which was remarkably spared (Figure 1A). As per panel design, PIK3CA mutations were detected in the coding region corresponding to the helical and kinase domains of the protein, with similar frequency (Figure 1B). PIK3CA mutations were mostly present at the three hot-spot codons (70 tumors carried p.Glu542; 120 p.Glu545; > 150 p.His1047). For TP53, 272 different mutations were observed; out of these, p.Arg175, p.Arg248 and p.Arg273 were observed in 15, 19 and 28 patients, respectively (total *n* = 60). All these mutated arginines were of mild to intermediate pathogenicity, located in the DBD domain, and reported within the Li-Fraumeni syndrome (NCBI, ClinVar database). The remaining TP53 mutated codons were affected in less than 10 cases each.

Mutations in either gene were found in 734 out of 1766 tumors with informative results (41.6%); 458 tumors (25.9%) had PIK3CA and 380 (21.5%) had TP53 mutations, corresponding to 62.4% and 51.8% of mutant tumors, respectively. The two genes were co-mutated in 104 cases (5.9% of all, 14.2% of mutant tumors). In 43 and 37 tumors more than one mutations were observed in PIK3CA and TP53, respectively. All TP53 and PIK3CA mutation data have been made publicly available at: http://www.hecog-images.gr/4adj/ngs/.

### Mutant TP53 and PIK3CA tumor phenotypes

**Figure 2:**
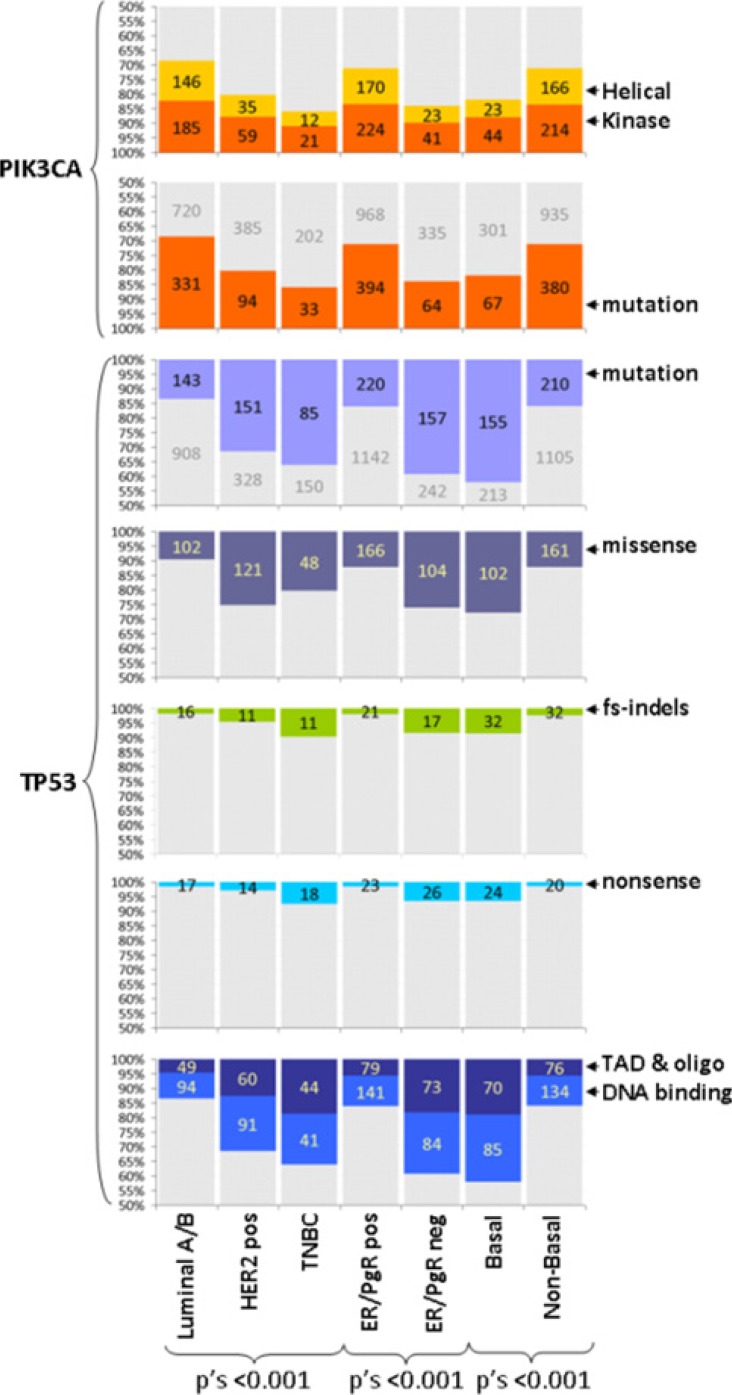
TP53 and PIK3CA mutation characteristics according to tumor subtypes Mutations are described for presence / absence and domain specificity as indicated. TP53 mutations are compared for mutation types (missense, frameshift indels, nonsense). Y-axes have been truncated at 50%. Numbers per category are shown. Grey parts in bars: complementary to the colored category. All mutations and their characteristics were related to ER/PgR status. Helical, kinase: mutations in the corresponding domains of PIK3CA; fs-indels: frameshift insertions / deletions; TAD, oligo: TP53 transactivation and oligomerization domains.

Luminal A and Luminal B tumors were examined as one group for the purposes of the present study, mainly because the concordance of defining these two subtypes with Ki67 immunohistochemistry (IHC) and with the PAM50 classifier is reported as low [31]. As expected [10], PIK3CA mutations were more common in Luminal A/B, overall in ER/PgR-positive and non-basal as compared to HER2-positive and TNBC, overall ER/PgR-negative and basal-like tumors; TP53 mutations were more common in HER2-positive and TNBC but infrequent in Luminal A/B, and similarly more common in ER/PgR-negative and basal-like tumors (Figure 2, Table S1). The distribution of TP53 mutation types was also subtype specific with more frameshift indels and nonsense mutations in TNBC, ER/PgR-negative and basal-like tumors, but these numbers per category were very small. The observed frameshifts in PIK3CA were not related to subtypes and ER/PgR positivity. Domain-specific mutations in both genes were also subtype- and ER/PgR-specific, whereby all tumor subtypes related to ER/PgR positivity were significantly more frequently mutated in the TP53 DNA binding domain than in the TAD and oligomerization domains; subtypes related to ER/PgR absence more frequently had more mutations in the helical than in the transactivation domain of the PIK3CA gene.

In line with the above mutation patterns concerning ER/PgR positivity, PIK3CA mutations were significantly more frequent in grade I tumors as compared to higher tumor grades; were frequent in lobular but rare in medullary carcinomas; and, were found in low proliferating tumors (Table S1). By contrast, TP53 mutations were detected with increasing frequency from grade I to II to III tumors; were rare in lobular but present in almost all medullary carcinomas; were positively associated with higher CEN17 median copies.

p53 immunopositivity was noticed in 848 of 1585 evaluable tumors (53.5%), followed the subtype-specific pattern of TP53 mutations (*p* = 0.001) and was positively associated with younger patient age (*p* = 0.009), basal phenotype (*p* = 0.029) and Ki67 labeling (*p* < 0.001).

The presence of TP53 mutations was strongly associated with p53 immunopositivity (*p* < 0.001) but the overall concordance between the two parameters was poor (Kappa = 0.18; Table S3). TP53 mutations were associated with p53 immunopositivity at a rate of 74.4%, which was higher for DNA binding domain (81%) and especially missense (89.7%) mutations; however, only half of p53 positive tumors carried TP53 mutations. Sixty-six percent of nonsense and frameshift mutations, generally prediciting for loss-of-function, were associated with negative p53 protein expression; again, the rate of such mutations within all p53 negative tumors was only 9.2%. The same pattern was observed for all major subtypes. These findings show that tumors with TP53 mutations are likely to be immunopositive for p53 protein but that p53 protein status does not predict for the presence of TP53 mutations, without any subtype specificity. A marginal association between p53 immunopositivity and PIK3CA mutation presence was also noticed (*p* = 0.046) (Table S3), with 52% of all PIK3CA, and 56% of PIK3CA missense mutant tumors demonstrating positive p53 protein status.

### TP53 mutations, PIK3CA mutations and p53 protein status affect disease-free survival (DFS)

Complete follow-up and mutation data were available for 1764 patients, with 3-year and 5-year DFS rates of 88.0% and 82.2%, respectively (Table 1); corresponding data were available for 1585 patients with informative p53 IHC, with 3-year and 5-year DFS rates of 88.3% and 82.8%, respectively.

**Table 1:** Patient demographics and clinicopathological parameters in the entire cohort and broken down into pre- and post-trastuzumab era trials

	Trial			
	Entire population	Pre-trastuzumab era trials (HE10/97 and ΗΕ10/00)	Post-trastuzumab era trials (ΗΕ10/05 and HE10/08)	*p*-value*
**Patients**				
*N*	1766	620	1.146	
**Age (years)**				
Mean (SD)	53.2 (11.5)	52.8 (11.2)	53.4 (11.7)	0.31
Median	52.9	52.4	53.3	
Min-Max	21-83	22-79	21-83	
**Tumor size in cm**				
Mean (SD)	2.9 (1.6)	3.3 (1.8)	2.7 (1.5)	< 0.001
Median	2.5	3	2.4	
Min-Max	0–15	0–15	0–11	
**Positive lymph nodes (*N*)**				
Mean (SD)	4.8 (6.4)	6.8 (6.9)	3.7 (5.9)	< 0.001
Median	2	4	2	
Min-Max	0–54	0–43	0–54	
**Ki67 (% of positive cells)**				
Mean (SD)	30.2 (26.7)	31.4 (23.7)	29.4 (28.3)	< 0.001
Median	20	25	19.5	
Min-Max	0–100	0–98	0–100	
**CEN17 copies (*N*)**				
Mean (SD)	2.4 (1.4)	2.6 (1.9)	2.3 (1.0)	0.007
Median	2	2.1	2	
Min-Max	1–18	1–18	1–11	
	***N*** **(%)**	***N*** **(%)**	***N*** **(%)**	
**Age (*N* = 1764)**				
≤ 50 years	724 (41.0)	270 (43.5)	454 (39.7)	0.12
> 50 years	1040 (59.0)	350 (56.5)	690 (60.3)	
**Menopausal status (*N* = 1764)**				
Postmenopausal	952 (54.0)	325 (52.4)	627 (54.8)	0.34
Premenopausal	812 (46.0)	295 (47.6)	517 (45.2)	
**Tumor size (*N* = 1763)**				
≤ 2 cm	639 (36.2)	174 (28.1)	465 (40.6)	< 0.001
> 2 cm	1124 (63.8)	445 (71.9)	679 (59.4)	
**Positive lymph nodes (*N* = 1764)**				
0–3	1057 (59.9)	253 (40.8)	804 (70.3)	< 0.001
≥ 4	707 (40.1)	367 (59.2)	340 (29.7)	
**Histological grade (***N* **= 1757)**				
I	113 (6.4)	29 (4.7)	84 (7.4)	0.083
II	792 (45.1)	282 (45.5)	510 (44.9)	
III-Undifferentiated	852 (48.5)	309 (49.8)	543 (47.8)	
**Histological type (*N* = 1764)**				
IC-NST	1442 (81.7)	472 (76.1)	970 (84.8)	< 0.001
Invasive lobular	157 (8.9)	60 (9.7)	97 (8.5)	
Mixed	83 (4.6)	48 (7.7)	35 (3.1)	
Other	82 (4.6)	40 (6.5)	42 (3.7)	
**Surgery (binary) (*N* = 1764)**				
MRM	1009 (57.2)	428 (69.0)	581 (50.8)	< 0.001
Other	755 (42.8)	192 (31.0)	563 (49.2)	
**Hormonotherapy (*N* = 1759)**				
No	403 (22.9)	121 (19.6)	282 (24.7)	0.017
Yes	1356 (77.1)	495 (80.4)	861 (75.3)	
**Radiotherapy (*N*= 1716)**				
No	414 (24.1)	128 (21.4)	286 (25.6)	0.058
Yes	1302 (75.9)	469 (78.6)	833 (72.4)	
**Subtypes entire cohort (*N* = 1765)**				
Luminal A	588 (33.3)	148 (23.9)	440 (38.4)	< 0.001
Luminal B	463 (26.2)	179 (28.9)	284 (24.8)	
Luminal-HER2	318 (18.0)	142 (23.9)	176 (15.4)	
HER2-Enriched	161 (9.1)	59 (9.5)	102 (8.9)	
TNBC	235 (13.3)	92 (14.8)	143 (12.5)	
**Subtypes entire cohort combined (*N* = 1765)**				
Luminal A/B	1051 (59.6)	327 (52.8)	724 (63.2)	<0.001
HER2-positive	235 (13.3)	92 (14.8)	143 (12.5)	
TNBC	479 (27.1)	201 (32.4)	278 (24.3)	
**Subtypes concordant (*N* = 1248)**				
Luminal A	506 (40.5)	121 (31.5)	385 (44.6)	< 0.001
Luminal B	388 (31.1)	141 (36.7)	247 (28.6)	
Luminal-HER2	150 (12.0)	55 (14.3)	95 (11.0)	
HER2-Enriched	91 (7.3)	32 (8.3)	59 (6.8)	
TNBC	113 (9.1)	35 (9.1)	78 (9.0)	
**Subtypes concordant combined (*N* = 1248)**				
Luminal A/B	894 (71.6)	262 (68.2)	632 (73.1)	0.13
HER2-positive	241 (19.3)	87 (22.7)	154 (17.8)	
TNBC	113 (9.1)	35 (9.1)	78 (9.0)	
**ER/PgR central (*N* = 1697)**				
Negative	312 (18.4)	115 (19.6)	197 (17.8)	0.36
Positive	1385 (81.6)	473 (80.4)	912 (82.2)	
**ER/PgR local (*N*= 1761)**				
Negative	399 (22.7)	151 (24.4)	248 (21.7)	0.19
Positive	1362 (77.3)	467 (75.6)	895 (78.3)	
**HER2 IHC central (*N* = 1692)**				
No overexpression	1459 (86.2)	502 (84.5)	957 (87.2)	0.13
Overexpression	233 (13.8)	92 (15.5)	141 (12.8)	
**HER2 IHC local (*N* = 1729)**				
No overexpression	1256 (72.6)	396 (67.1)	860 (75.5)	< 0.001
Overexpression	473 (27.4)	194 (32.9)	279 (24.5)	
**HER2 status central (*N* = 1707)**				
Negative	1305 (76.4)	444 (74.1)	861 (77.7)	0.096
Positive	402 (23.6)	155 (25.9)	247 (22.3)	
**CK5 central (*N*= 1689)**				
Negative	1452 (86.0)	523 (89.7)	929 (84.0)	0.001
Positive	237 (14.0)	60 (10.3)	177 (16.0)	
**EGFR central (*N* = 1690)**				
Negative	1404 (83.1)	487 (83.4)	917 (82.9)	0.80
Positive	286 (16.9)	97 (16.6)	189 (17.1)	
**Basal (***N* **= 1683)**				
Basal	368 (21.9)	106 (18.2)	262 (23.8)	0.009
Non-Basal	1315 (78.1)	475 (81.8)	840 (76.2)	
**RandomGroup**				
E-CMF	86 (4.8)	86 (13.8)	0 (0.0)	< 0.001
E-CMF-Doc	182 (10.4)	0 (0.0)	182 (15.8)	
E-CMF-T	199 (11.2)	0 (0.0)	199 (17.4)	
E-T-CMF	1059 (60.0)	294 (47.4)	765 (66.8)	
ET-CMF	240 (13.6)	240 (38.8)	0 (0.0)	
**Survival data**				
Median FU in months	72.5	118.4	65.6	
N of valid cases	1764	620	1144	< 0.001
Deaths (N)	278	181	97	
Event-free at 3 years	95.4	93.8	96.2	
Event-free at 5 years	89.3	85.2	91.7	
Relapses (N)	389	225	164	< 0.001
Event-free at 3 years	88.0	82.8	90.9	
Event-free at 5 years	82.2	74.5	86.5	

All patients had informative NGS data.

Notes: MRM: modified radical mastectomy; FU: follow-up; N: number; IC-NST: invasive carcinoma of non-specific type; IHC: immunohistochemistry.

* Comparison of variable categories in the pre- and post- trastuzumab era series.

**Figure 3:**
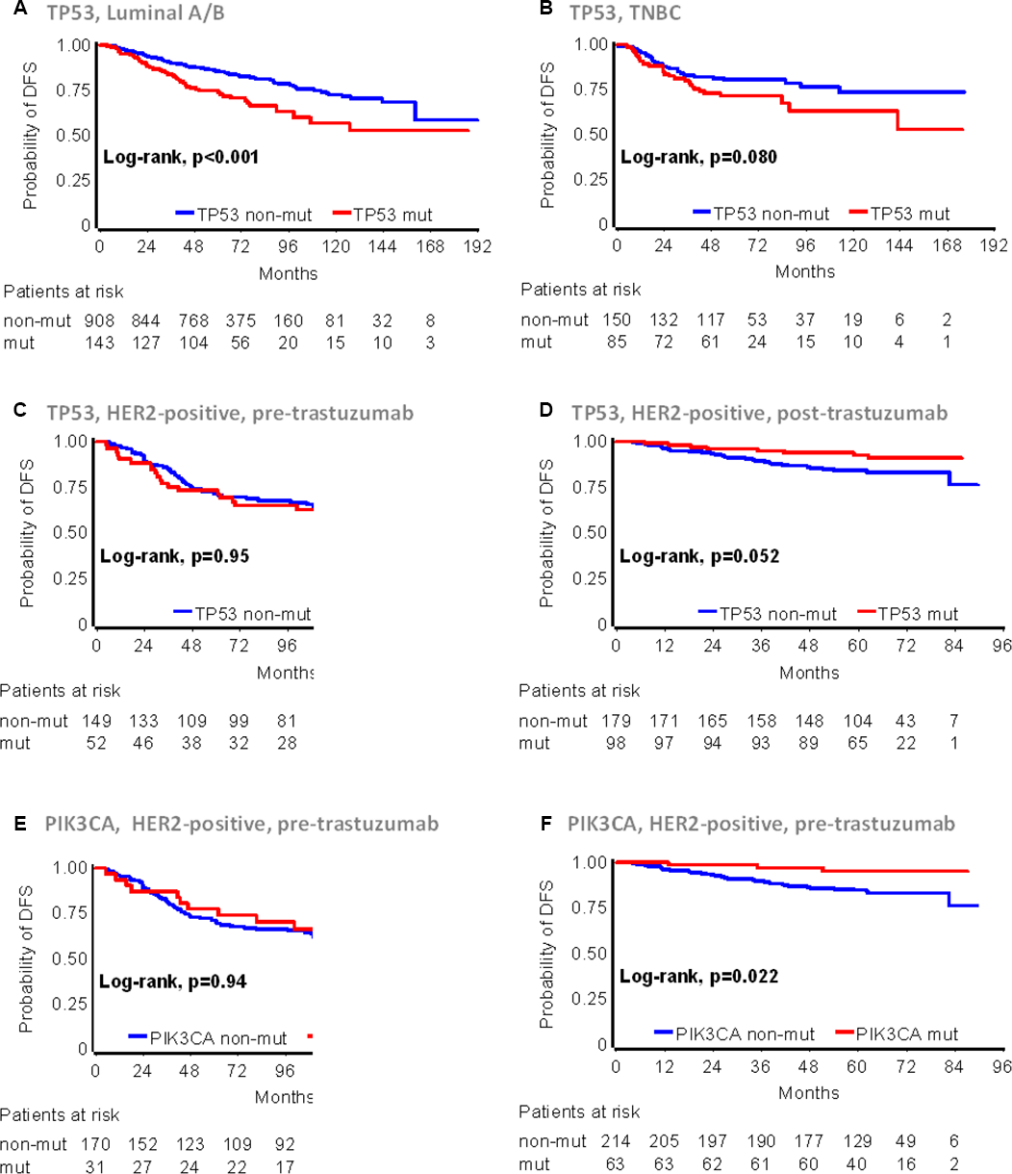
TP53 and PIK3CA mutation effects on early breast cancer patient DFS according to main disease subtypes and trastuzumab treatment The presence of TP53 mutations was unfavorable in Luminal A/B (**A**) and TNBC (**B**), indifferent in HER2-positive patients who were treated with anthracyclines only in the pre-trastuzumab era (**C**), but favorable in trastuzumab treated HER2-positive patients (**D**). (**E**) and (**F**): PIK3CA mutations in HER2-positive, non-trastuzumab and trastuzumab treated patients were similar to those described for TP53 mutations in (C) and (D), respectively.

Univariate Cox results for all mutation and IHC markers with respect to patient DFS are shown in Table 2. Among the 1784 patients with informative NGS data, patients with TP53 mutations had statistically significantly worse DFS (HR = 1.34; 95% CI 1.07–1.68; *p* = 0.011). Among the three main subtypes, TP53 mutations were strongly unfavorable in patients with Luminal A/B tumors, which constituted the largest subgroup, albeit with the lowest rate of this genomic alteration in the entire cohort (Figure 3A) and marginally unfavorable in patients with TNBC (Figure 3B). Grouping of Luminal A/B tumors for mutation analyses was justified by the lack of interaction between TP53 mutations and these two subtypes, when examined separately (Table S4). By contrast, HER2-positive patients with TP53 mutant tumors showed a trend for longer DFS than those without (HR = 0.69; 95% CI 0.45 – 1.06; *p* = 0.092). Because trastuzumab was administered only in two of the analyzed studies (HE10/05 and HE10/08) we next assessed TP53 mutations separately in the pre- [32, 33] and post-trastuzumab [34] era. In the pre-trastuzumab era, TP53 mutations did not affect DFS (Figure 3C). Among patients who received trastuzumab, those with TP53 mutations fared marginally better as compared to those without (Figure 3D).

**Table 2:** Univariate cox regression analysis for TP53 and PIK3CA mutation (MUT) and for p53 protein expression by IHC against disease-free survival

Parameter	*N* patients	*N* events	HR	95% CI	Wald's *p*
	**ENTIRE COHORT**				
**TP53 mutations**					
YES vs. NO	380 vs. 1386	101 vs. 288	1.34	1.07–1.68	0.011
**TP53 mutation type**					0.014
missense vs. none	267 vs. 1386	78 vs. 288	1.51	1.17–1.94	0.001
fs-indels vs. none	64 vs. 1386	13 vs. 288	0.99	0.57–1.73	0.97
nonsense vs. none	49 vs. 1386	10 vs. 288	0.96	0.51–1.8	0.89
**TP53 mutated domains**					0.03
DBD vs. none	225 vs. 1386	63 vs. 288	1.42	1.08–1.86	0.012
other vs. none	155 vs. 1386	38 vs. 288	1.23	0.88–1.73	0.23
**PIK3CA mutations**					
YES vs. NO	458 vs. 1308	89 vs. 300	0.83	0.65–1.05	0.12
**PIK3CA mutated domains**					0.12
kinase vs. none	265 vs. 1308	57 vs. 300	0.94	0.71–1.24	0.65
helical vs. none	193 vs. 1308	32 vs. 300	0.68	0.48–0.99	0.042
**TP53 or PIK3CA mutations**					
YES vs. NO	734 vs. 1032	159 vs. 230	0.97	0.79–1.19	0.78
**Mutation breakdown**					0.006
PIK3CA only vs. none	354 vs. 1032	58 vs. 230	0.71	0.53–0.94	0.018
TP53 only vs. none	276 vs. 1032	70 vs. 230	1.17	0.90–1.53	0.25
both vs. none	104 vs. 1032	31 vs. 230	1.42	0.97–2.07	0.068
**p53 IHC 10% cut off**					
≥ 10% vs. <10%	848 vs. 737	176 vs. 162	0.95	0.76–1.17	0.61
	**LUMINAL A/B**				
**TP53 mutations**					
YES vs. NO	143 vs. 908	47 vs. 171	1.86	1.34–2.57	< 0.001
**TP53 mutation type**					< 0.001
missense vs. none	102 vs. 908	34 vs. 171	1.90	1.32–2.75	< 0.001
fs-indels vs. none	24 vs. 908	6 vs. 171	1.23	0.54–2.78	0.62
nonsense vs. none	17 vs. 908	7 vs. 171	2.71	1.27–5.78	0.010
**TP53 mutated domains**					< 0.001
DBD vs. none	93 vs. 908	30 vs. 171	1.75	1.18–2.58	0.005
other vs. none	50 vs. 908	17 vs. 171	2.09	1.27–3.44	0.004
**PIK3CA mutations**					
YES vs. NO	331 vs. 720	64 vs. 154	0.88	0.66–1.18	0.39
**PIK3CA mutated domains**					0.56
kinase vs. none	185 vs. 720	38 vs. 154	0.95	0.66–1.35	0.76
helical vs. none	146 vs. 720	26 vs. 154	0.80	0.53–1.21	0.29
**TP53 or PIK3CA mutations**					
YES vs. NO	423 vs. 628	92 vs. 126	1.07	0.82–1.41	0.61
**Mutation breakdown**					< 0.001
PIK3CA only vs. none	280 vs. 628	45 vs. 126	0.78	0.55–1.08	0.13
TP53 only vs. none	92 vs. 628	28 vs. 126	1.58	1.05–2.38	0.030
both vs. none	51 vs. 628	19 vs. 126	1.99	1.23–3.23	0.005
**p53 IHC 10% cut off**					
≥ 10% vs. < 10%	485 vs. 456	96 vs. 94	0.93	0.7–1.24	0.65
	**TNBC**				
**TP53 mutations**					
YES vs. NO	85 vs. 150	27 vs. 32	1.58	0.94–2.63	0.083
**TP53 mutation type**					0.044
missense vs. none	47 vs. 150	19 vs. 32	2.16	1.22–3.82	0.008
fs-indels vs. none	20 vs. 150	5 vs. 32	1.22	0.48–3.14	0.68
nonsense vs. none	18 vs. 150	3 vs. 32	0.71	0.22–2.32	0.57
**TP53 mutated domains**					0.14
DBD vs. none	41 vs. 150	15 vs. 32	1.86	1.01–3.45	0.047
other vs. none	44 vs. 150	12 vs. 32	1.32	0.68–2.57	0.41
**PIK3CA mutations**					
YES vs. NO	33 vs. 202	10 vs. 49	1.27	0.64–2.51	0.50
**PIK3CA mutated domains**					0.068
kinase vs. none	21 vs. 202	9 vs. 49	2.00	0.98–4.08	0.056
helical vs. none	12 vs. 202	1 vs. 49	0.29	0.04–2.13	0.22
**TP53 or PIK3CA mutations**					
YES vs. NO	103 vs. 132	31 vs. 28	1.47	0.88–2.46	0.14
**Mutation breakdown**					0.29
PIK3CA only vs. none	18 vs. 132	4 vs. 28	1.01	0.36–2.89	0.98
TP53 only vs. none	70 vs. 132	21 vs. 28	1.47	0.84–2.6	0.18
both vs. none	15 vs. 132	6 vs. 28	2.10	0.87–5.09	0.10
**p53 IHC 10% cut off**					
≥ 10% vs. < 10%	113 vs. 102	30 vs. 25	1.09	0.64–1.86	0.74
	**HER2-positive, pre-trastuzumab**				
**TP53 mutations**					
YES vs. NO	52 vs. 149	19 vs. 55	1.01	0.6–1.7	0.97
**TP53 mutation type**					0.48
missense vs. none	42 vs. 149	18 vs. 55	1.28	0.75–2.19	0.36
fs-indels vs. none	6 vs. 149	1 vs. 55	0.62	0.12–3.22	0.57
nonsense vs. none	4 vs. 149	0 vs. 55	0.20	0.01–3.72	0.28
**TP53 mutated domains**					0.46
DBD vs. none	31 vs. 149	13 vs. 55	1.27	0.7–2.33	0.43
other vs. none	21 vs. 149	6 vs. 55	0.69	0.29–1.62	0.38
**PIK3CA mutations**					
YES vs. NO	31 vs. 170	12 vs. 62	1.03	0.55–1.9	0.94
**PIK3CA mutated domains**					0.96
kinase vs. none	17 vs. 170	7 vs. 62	1.10	0.5–2.41	0.81
helical vs. none	14 vs. 170	5 vs. 62	0.93	0.38–2.33	0.89
**TP53 or PIK3CA mutations**					
YES vs. NO	72 vs. 129	27 vs. 47	1.01	0.63–1.62	0.97
**Mutation breakdown**					<0.99
PIK3CA only vs. none	20 vs. 129	8 vs. 47	1.01	0.48–2.13	0.99
TP53 only vs. none	41 vs. 129	15 vs. 47	1.00	0.55–1.79	0.99
both vs. none	11 vs. 129	4 vs. 47	1.07	0.38–2.95	0.91
**p53 IHC 10% cut off**					
≥ 10% vs. < 10%	92 vs. 86	38 vs. 23	1.71	1.02–2.87	0.043
	**HER2-positive, post-trastuzumab**				
**TP53 mutations**					
YES vs. NO	98 vs. 179	8 vs. 30	0.47	0.22–1.02	0.06
**TP53 mutation type**					0.45
missense vs. none	76 vs. 179	7 vs. 30	0.56	0.25–1.27	0.16
fs-indels vs. none	13 vs. 179	1 vs. 30	0.72	0.13–3.87	0.70
nonsense vs. none	10 vs. 179	0 vs. 30	0.28	0.02–4.89	0.38
**TP53 mutated domains**					0.16
DBD vs. none	60 vs. 179	5 vs. 30	0.47	0.18–1.23	0.12
other vs. none	38 vs. 179	3 vs. 30	0.46	0.14–1.5	0.20
**PIK3CA mutations**					
YES vs. NO	63 vs. 214	3 vs. 35	0.28	0.09–0.9	0.032
PIK3CA mutated domains					0.18
kinase vs. none	42 vs. 214	3 vs. 35	0.47	0.15–1.45	0.19
helical vs. none	21 vs. 214	0 vs. 35	0.14	0.01–2.37	0.17
**TP53 or PIK3CA mutations**					
YES vs. NO	134 vs. 143	9 vs. 29	0.31	0.15–0.66	0.002
**Mutation breakdown**					0.026
PIK3CA only vs. none	36 vs. 143	1 vs. 29	0.13	0.02–0.92	0.042
TP53 only vs. none	71 vs. 143	6 vs. 29	0.39	0.16–0.95	0.038
both vs. none	27 vs. 143	2 vs. 29	0.34	0.08–1.44	0.14
**p53 IHC 10% cut off**					
≥ 10% vs. < 10%	160 vs. 92	12 vs. 20	0.31	0.15–0.64	0.002

Notes: TAD: transactivation domain; DBD: DNA binding domain; TETRA: oligomerization domain; IHC: Immunohistochemistry; Luminal A/B: ER/PgR positive, HER2 negative.

Domain specificity of mutations was associated with DFS in Luminal A/B and TNBC, while it was without effect in HER2-positive patients (Table 2). Analysis of the three most commonly mutated arginines (codons 175, 248 and 273) did not produce meaningful results due to the small sample size; it should be noticed however, that among the 19 patients with TP53 codon 248 mutations, only 1 patient with p.Arg248Gln relapsed after 80 months of treatment. The tumor was initially called TNBC, but was classified as Luminal B upon central testing. Immunopositivity for p53 significantly affected DFS in patients with HER2-positive tumors only, in a way similar to that of TP53 mutations; HER2-positive, p53 IHC positive tumors were associated with increased risk for relapse in the pre-trastuzumab era, while the same phenotype conferred favorable DFS in the post-trastuzumab era trials. No effect was observed for PIK3CA mutations when they were examined in the entire cohort including all subtypes. The presence of PIK3CA mutations resulted in favorable DFS for HER2-positive patients in the post-, but was without effect in the pre-trastuzumab era trials (Figure 3E and 3F); PIK3CA mutations and their domain specificity were without effect in the Luminal A/B and TNBC groups. TP53 and PIK3CA co-mutated tumors conferred increased risk for relapse in Luminal A/B patients only, but not in the other subtypes. Finally, among HER2-positive, trastuzumab-treated patients, the presence of either TP53 or PIK3CA mutations was associated with favorable DFS; by contrast, the presence of either mutant gene predicted for worse DFS in HER2-positive patients in the post-trastuzumab era trials (Table 2).

### TP53 mutations and p53 protein status interacted with trastuzumab benefit

The HER2-subtype-specific diverse effects of TP53 and PIK3CA mutations, as well as those of p53 protein expression, prompted us to further investigate a possible role of these characteristics in predicting trastuzumab benefit. For this purpose, the pre-trastuzumab studies were excluded from the analyses, due to the respective much longer follow-up as compared to the post-trastuzumab studies (Table 1). Trastuzumab had been administered according to local HER2-positive diagnosis over a period of 7 years. Upon retrospective testing, 50 tumors were centrally characterized as HER2-positive although they were HER2-negative when locally assessed and those patients had therefore not received trastuzumab. For testing putative interactions between mutations or p53 protein expression and trastuzumab, we compared this 50-patient, HER2-positive non-treated group against 177 patients with locally and centrally HER2-positive tumors that had been treated with the drug. In contrast to the pre- and post-trastuzumab HER-positive groups that did not differ with respect to ER/PgR status, the non-treated 50-patient subset was richer in ER/PgR-positive tumors than the HER2-concordant subset (Supplementary File S1, Part A).

**Figure 4:**
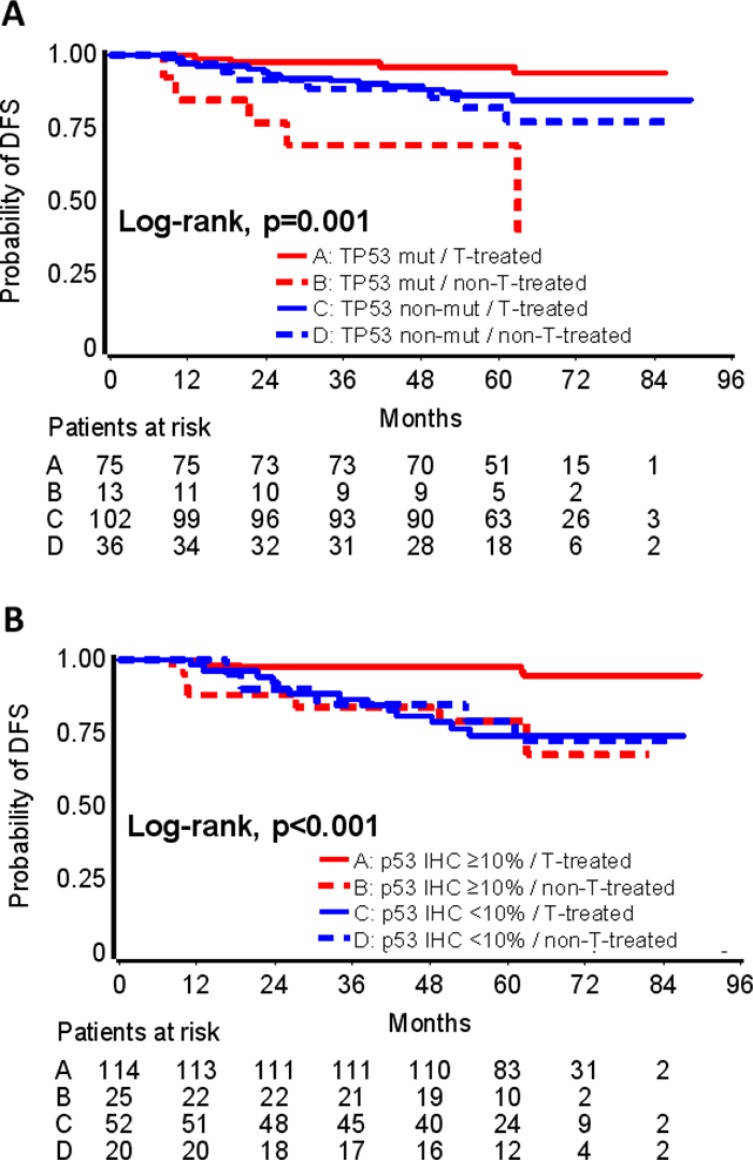
TP53 mutations predictive for trastuzumab benefit Interactions between TP53 mutations and trastuzumab (**A**), and between p53 protein expression and trastuzumab (**B**) are shown. Trastuzumab (T) treated patients with TP53 mutations (A) or p53 positive protein expression by IHC (B) fared best; patients with the same TP53 tumor properties, who were not treated with trastuzumab, fared worst.

By comparing DFS in the above patient subsets, TP53 mutations interacted with trastuzumab (interaction *p* = 0.017) and in a similar way with p53 immnopositivity (interaction *p* = 0.015) (Table 3). In particular, among patients with TP53 mutations (Figure 4A) or p53 immunopositivity (Figure 4B), those treated with trastuzumab had longer DFS than those not treated; in addition, the same TP53 markers conferred longer DFS in patients treated with trastuzumab but not in those who were not treated with the drug.

**Table 3:** Interaction testing between study variables and trastuzumab (T) treatment in HER2-positive patients

Parameter	*N* patients	*N* events	HR	95% CI	Wald's*p*^
**TP53 mutations (MUT)**					0, 017
TP53 MUT, YES vs. NO @ T-treated*	75 vs. 102	4 vs. 15	0.35	0.12–1.06	
TP53 MUT, YES vs. NO @ non-T-treated**	13 vs. 36	5 vs. 7	2.43	0.77–7.68	
T-treated vs. non-T-treated @ TP53 MUT, NO	102 vs. 36	15 vs. 7	0.69	0.28–1.68	
T-treated vs. non-T-treated @ TP53 MUT, YES	75 vs. 13	4 vs. 5	0.10	0.03–0.37	
**p53 IHC (10% cut-off)**					0, 015
p53 IHC, ≥ 10% vs. < 10% @ T-treated	114 vs. 52	5 vs. 13	0.15	0.05–0.42	
p53 IHC, ≥ 10% vs. < 10% @ non-T-treated	25 vs. 20	6 vs. 5	1.05	0.32–3.46	
T-treated vs. non-T-treated @ p53 IHC, < 10%	52 vs. 20	13 vs. 5	1.01	0.36–2.82	
T-treated vs. non-T-treated @ p53 IHC, ≥ 10%	114 vs. 25	5 vs. 6	0.14	0.04–0.47	
**PIK3CA mutations (MUT)**					0, 25
PIK3CA MUT, YES vs. NO @ T-treated	37 vs. 140	2 vs. 17	0.43	0.1–1.87	
PIK3CA MUT, YES vs. NO @ non-T-treated	13 vs. 37	4 vs. 8	1.31	0.39–4.36	
T-treated vs. non-T-treated @ PIK3CA MUT, NO	140 vs. 37	17 vs. 8	0.46	0.2–1.07	
T-treated vs. non-T-treated @ PIK3CA MUT, YES	37 vs. 13	2 vs. 4	0.15	0.03–0.83	

Notes: T: trastuzumab; IHC: Immunohistochemistry;

^ interaction p;

* HER2 status positive upon local and central testing, treated with trastuzumab

** HER2 status negative upon local, positive upon central testing, not treated with trastuzumab.

In contrast to TP53 mutations and protein expression, no interaction was identified for PIK3CA mutations with trastuzumab when comparing the same patient groups (Table 3).

### TP53 mutations and p53 protein status as subtype-specific independent prognosticators

**Figure 5:**
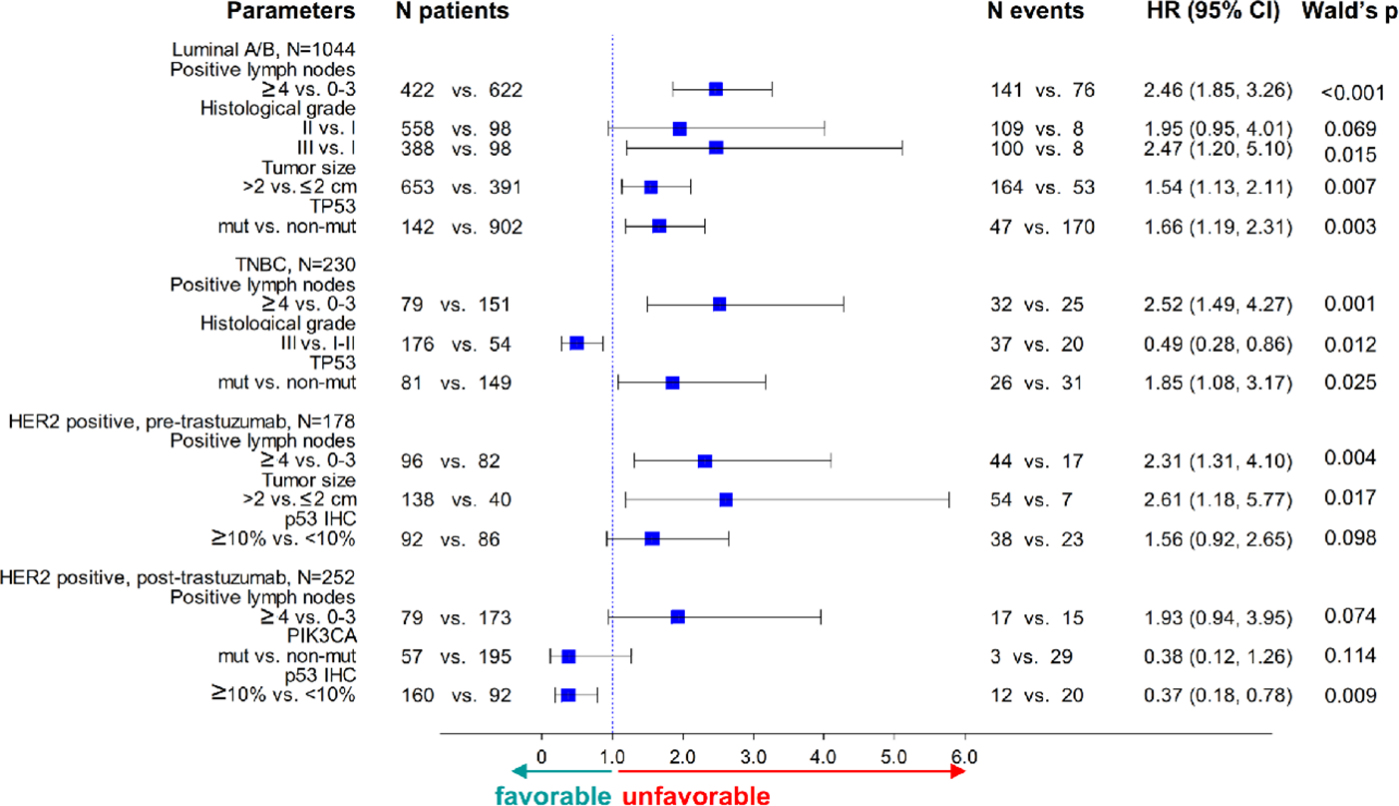
Forest plots for multivariable analyses in the entire cohort TP53 mutations and p53 protein expression were included in all models. p53 IHC positivity was unfavorable in HER2-positive patients not treated but favorable in those treated with trastuzumab.

Based on the subtype-specific impact of TP53 and PIK3CA mutations, and of p53 protein expression, multivariate analyses were performed in the three major subsets of breast cancer patients, i.e., Luminal A/B, TNBC and HER2-positive, the latter in the pre- and post-trastuzumab era. The impact of TP53 mutations, PIK3CA mutations, p53 protein expression and their interactions with trastuzumab on patient DFS was adjusted for standard clinicopathological parameters (Table S5) in three different settings, (i) in the entire patient population by local pathology subtyping, upon which the administration of hormone therapy and trastuzumab was based (Figure 5); (ii) in centrally typed HER2-positive cases, for which the interactions with trastuzumab were identified, as described above (Table S6); and, (iii) in cases with concordant subtyping upon local and central testing (Table S7). The latter approach was undertaken as a more stringent validation of the above findings concerning TP53 mutations and p53 protein expression. Discordance rates according to ER/PgR and HER2 positivity are shown in Table S8.

TP53 mutations and their domain specificity were significantly unfavorable in Luminal A/B and TNBC in the entire cohort (Figure 5, Table S9) and retained their significance in Luminal A/B concordant patients (Table S7 and Table S10). For HER2-positive patients in the post-trastuzumab era, the interaction between TP53 mutations or p53 protein expression and trastuzumab was independently significant for DFS, whereby positivity for these markers conferred decreased risk for relapse in trastuzumab-treated patients. In the pre-trastuzumab era trials, TP53 mutations were either not retained in the model or, if significant, they were associated with a higher risk for relapse, the opposite effect than the one observed in the post-trastuzumab era trials.

## DISCUSSION

The primary objective of the present study was to investigate important associations of tumor TP53 and PIK3CA mutations with clinical outcome in patients with early breast cancer enrolled in four prospective clinical studies. Following targeted next generation sequencing genotyping in 1766 FFPE DNA samples, PIK3CA and TP53 gene mutations were found in 25.9% and 21.5% of the patients, respectively, percentages that are remarkably similar to those reported for breast cancer in the Catalog of Somatic Mutations in Cancer (COSMIC, 26% and 23%, respectively) [35]. Additionally, in line with the published literature [17, 36], the majority of mutations in both genes were missense. Most of the TP53 mutations were found in the DNA binding domain, as has also been reported by others [16], while PIK3CA mutations in the helical and kinase domains were found at the expected incidence for the three hot-spot codons, as has been repetitively reported [37].

As previously stated, TP53 mutations have been linked to reduced survival of patients with breast cancer compared to wild-type TP53. As we are deepening our knowledge in tumor cell biology, it is becoming increasingly evident that the properties of specific mutants with regard to tumor progression and their clinical applications remain unclear [35, 38]. In the present study no specific effect for missense, nonsense or frameshift TP53 mutations was revealed. In line with earlier evidence [39], we have also demonstrated the unfavorable effect of DNA binding domain mutations. As suggested [40], however, not all of these mutations have the same impact on patient outcome. For example, as shown here, p.Arg248Gln mutations may confer favorable prognosis. This is in contrast to the unfavorable prognostic effect previously assigned to the same mutation [13], the reason probably being that patient subsets with specific mutated codons are small within individual studies. Nevertheless, since the present study included Greek patients only, this type of information is noteworthy because notable differences in the spectrum or prognostic impact of mutations, especially in TP53, have been reported among different ethnic groups or geographical regions, possibly due to the link with environmental mutagens [41, 42]. Such differences may be crucial when evaluating results of multinational clinical trials, investigating novel targeted agents.

With respect to disease characteristics, this study confirms the prevalence of TP53 mutations in ER/PgR-negative and that of PIK3CA mutations in ER/PgR-positive tumors [10]. In accordance with PIK3CA mutations developing in a hormone receptor rich environment, these mutations were more frequently observed in lobular than in ductal carcinomas, a finding that has been reported by others, as well [36]. However, contrary to the published literature [13, 43] and the predominant view that the presence of TP53 mutations are more frequent in high-grade, large-size, node-positive breast cancers, we did not observe, with the exception of histological grade, a significant association between TP53 mutations and menopausal status, tumor size or nodal status. This discrepancy may well be due to a selection bias in patient populations of prospective clinical trials (as in the case of the present study), in which only patients with intermediate or high-risk cancers were included, as opposed to unselected series [43].

The above subtype specificity of TP53 and PIK3CA mutations is reflected in the impact of these mutations on patient DFS. In a thorough study of the spectrum of TP53 mutations, Silwal-Pandit et al. [15] obtained 1420 tumor samples from the METABRIC cohort [43] and sequenced all coding exons of the TP53 gene. By using the PAM50 classifier, they concluded that TP53 mutations were associated with worse prognosis in patients with Luminal B, HER2-enriched and normal-like tumors, but not in patients with Luminal A or Basal-like tumors. In a partial overlap with this report, herein we show that for tumors subtyped with IHC and HER2 FISH, where needed, TP53 mutations were strong independent adverse prognosticators in patients with Luminal A/B and TNBC, but not in patients with HER2-positive tumors. The observed differences most probably reflect the approximately 40% discrepancy rate between the two subtyping approaches [31, 44]. In the case of Luminal A/B tumors, where TP53 mutations were strongly unfavorable, our data seem to support that TP53 mutation dependence, once established, provides a more severe pro-oncogenic activity compared to the co-existing hormone dependence [13, 35]. As shown, TP53 mutations were unfavorable in ER/PgR-positive HER2-negative tumors irrespectively of Luminal A and Luminal B distinction. Although the rate of TP53 mutations in Luminal A/B tumors was the lowest among the major breast cancer subtypes, the number of patients with such mutations is considerably large. In the context of the world-wide applied clinical subtyping, the adverse prognostic effect of TP53 mutations in this large group of patients is a novel finding that merits further clinical investigation.

In contrast to TP53 mutations, no prognostic role was revealed here for p53 protein expression in the entire cohort or in Luminal A/B and TNBC patients, in line with the CALGB 9344 study [25] that used the anti-p53 DO7 clone, as well. These findings cannot however be compared to the other two existing studies reporting on the poor prognosis of p53 immunopositive luminal tumors [23, 24], because of different IHC scoring systems and antibodies used.

TP53 mutations have been associated with response to various treatments of breast cancer [12]. Patients in the present study had been treated with adjuvant regimens comprised of an anthracycline, taxanes and CMF. Contradictory results regarding TP53 mutations and response to anthracycline-based chemotherapy, mainly in the neo-adjuvant setting, have been published, with some studies indicating chemosensitivity with improved pathological complete response rates [45, 46], while others are suggestive of chemoresistance [47–49]. Associations of TP53 mutations with response to anti-HER2 treatments have been reported mainly in the neo-adjuvant setting [50–52]. The present study is perhaps the first to indirectly show a favorable predictive effect of TP53 mutations and, intriguingly, p53 immnopositivity for trastuzumab benefit in the adjuvant setting. Herein, we compared the outcome of HER2-positive patients that were either treated or not with trastuzumab, although the latter did not a priori comprized a specific control group. Based on the present findings, p53 dysfunction may favor trastuzumab-specific responses in the adjuvant setting. Clearly, these findings are hypothesis generating. If further validated, the potential predictive role of p53-pathway aberrations for trastuzumab benefit may have important implications in the assessment of patients with HER2-positive operable disease.

PIK3CA mutational status has also been proposed as a potential marker of trastuzumab response. Preclinical studies indicate that PIK3CA mutations activate signaling downstream of HER2, resulting in relative resistance to HER2-targeted agents, including trastuzumab and lapatinib [37, 53–55]. Regarding the role of PIK3CA mutations in patients with HER2-positive operable or metastatic disease treated with anti-HER2 agents, at present, existing data are inconclusive. In the FinHER trial [56], patients with PIK3CA mutations had a better prognosis only during the first three years from randomization, but such mutations were not predictive for trastuzumab benefit [36]. Similarly, in the NSABP B31 trial [57], PIK3CA mutations were not predictive for trastuzumab benefit in the HER2-Enriched subtype, as defined by the PAM50 classifier [58]. On the other hand, genomic data from neo-adjuvant trials with anti-HER2 agents, strongly indicate that the presence of PIK3CA mutations predicted for poor pathological complete response and compromized survival [59–63]. In our series, PIK3CA mutations were not prognostic in Luminal A/B and TNBC patients, did not interact with trastuzumab treatment, while they lost their prognostic significance in trastuzumab-treated patients upon multivariate analysis. These data are in line with the adjuvant studies cited above. Possible points to be addressed in future studies in the adjuvant setting with respect to evaluating the importance of PIK3CA mutations, are larger numbers of PIK3CA only mutant tumors, which, as shown here, behave differently than those co-mutated with TP53; perhaps studying mutations with proven oncogenic potential among all mutations identified with next generation sequencing platforms; and, ensuring statistical power for PIK3CA mutant patient subsets, such as those with HER2-positive tumors.

In conclusion, in the present series, a definite prognostic/predictive role of PIK3CA mutations could not be demonstrated. On the other hand, TP53 mutations may be helpful in predicting poor prognosis in early breast cancer patients with Luminal A/B tumors and probably with TNBC, while immunopositivity for p53 protein may be predictive for adjuvant trastuzumab benefit. These findings, especially the potential value of positive p53 protein status, by the widely used IHC method, as a predictive marker for trastuzumab benefit, are worth validating in independent large prospective studies.

## MATERIALS AND METHODS

**Figure 6:**
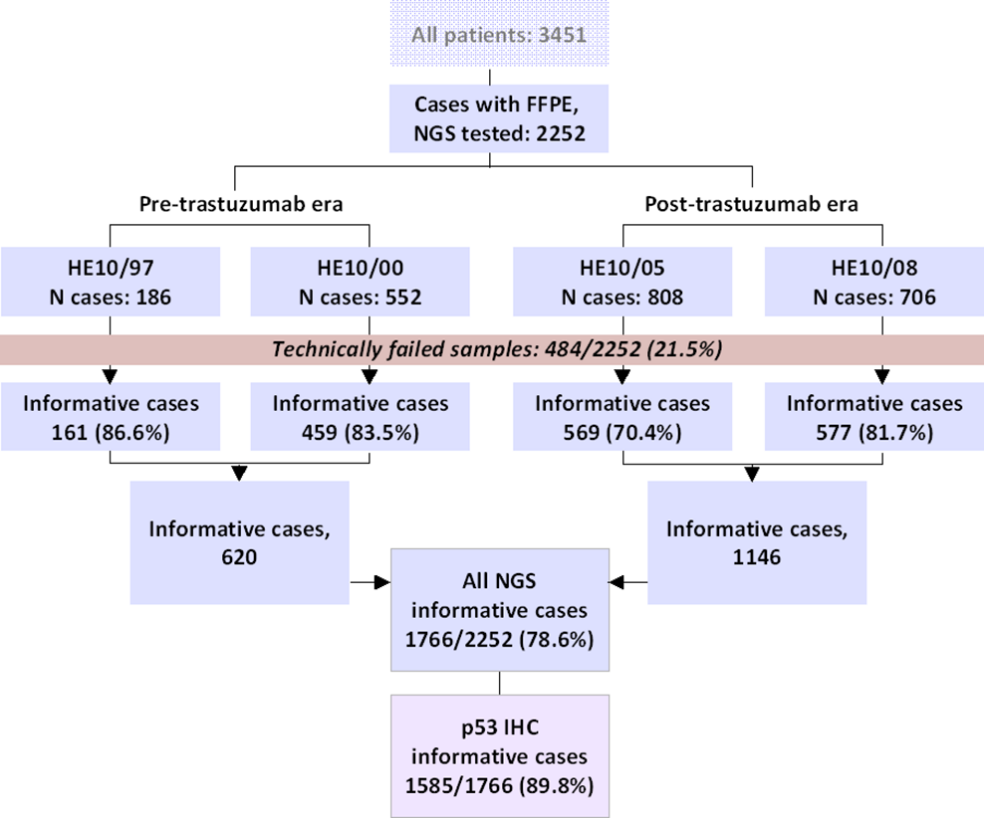
REMARK chart All samples examined in this study have yielded informative NGS results. Immunohistochemistry (IHC) for p53 protein expression was applied on available tissues in the majority of the NGS informative samples.

Tumor tissue material was examined from 2252 patients out of 3451 who had been diagnosed between 1997 and 2010 with operable breast cancer and had been treated with adjuvant chemotherapy (anthracyclines – taxanes) in the setting of four prospective clinical trials by the Hellenic Cooperative Oncology Group (HeCOG) (Figure 6). The basic trial characteristics are shown in Table S11. In HE10/97 [32] and HE10/00 [33] trastuzumab was not administered (pre-trastuzumab era). In HE10/05 [34] and HE10/08 (manuscript in preparation) trastuzumab was administered sequentially for one year after the completion of chemotherapy (post-trastuzumab era). Patients had provided written consent for the use of their biologic material for research purposes and the study was approved by the Bioethics Committee of the Aristotle University of Thessaloniki School of Health Sciences, Faculty of Medicine (#77/10June2014) and by the Institutional Review Board of the Papageorgiou Hospital of Thessaloniki (#725/10May2013). Paraffin blocks were collected retrospectively for HE10/97 and prospectively for the other three trials. The distribution of patients and tumors per clinical study and basic demographic, clinicopathological, follow-up and outcome data are shown in Table 1.

Tumors had been routinely diagnosed in local pathology laboratories, where they had also been evaluated for ER/PgR/HER2 protein expression with immunohistochemistry (IHC) and, in HE10/05 and in HE10/08, with FISH or CISH for HER2 IHC 2+ cases. Patients were stratified according to local testing for receiving hormone treatment (all trials) and trastuzumab (HE10/05 and HE10/08). For the purpose of the present study, based on local testing results, tumors were classified as Luminal A/B for combined ER/PgR positivity in the absence of HER2 protein overexpression and/or gene amplification; as triple negative (TNBC) if ER/PgR/HER2 negative; and, as HER2-positive, if HER2 IHC 3+ and/or HER2 FISH positive, independently of ER/PgR status.

The available paraffin blocks (routinely processed formalin-fixed paraffin-embedded, FFPE) were also centrally processed at the Laboratory of Molecular Oncology (Hellenic Foundation for Cancer Research/Aristotle University of Thessaloniki, Thessaloniki, Greece). Processing involved thorough central histological review by three experienced pathologists (S.L., M.B., A.B.), parameter recording and marking tumor-dense areas for macro-dissection and tissue microarray (TMA) construction. Low-density TMA blocks carried 2 × 1.5 mm cores from different areas of each tumor. All tumors were clinically subtyped with ER/PgR/HER2/Ki67 IHC and FISH, as previously described [64] with the Ki67 cut-off at 14% for distinguishing between Luminal A and Luminal B tumors. In addition, cytokeratin-5 (CK5) and epidermal growth factor receptor (EGFR) IHC was applied for typing basal-like carcinomas, with 1% cut-off for positivity, as suggested [65].

### DNA extraction and tissue processing for next generation sequencing (NGS)

Paraffin tumor sections were processed for DNA extraction upon manual macro-dissection in order to enrich samples for tumor DNA, as previously described [66] or, in the case of HE10/97, from TMA cores, 1.5 mm in diameter (5 × 8 um sections, 2 cores per tumor). Tumor cell content (TCC) was assessed as an approximate metric for tumor DNA in the extracted samples, corresponding to tumor nuclei vs. all nuclei in the areas marked for macro-dissection and on the TMA cores. The majority of samples (52%) had TCC ≥ 50% but samples with as low as 15% TCC were also processed, since it proved possible to orthogonally validate variants in such samples [67]. DNA was extracted with magnetic beads (VERSANT Tissue Prep Kit, Siemens Healthcare, Erlangen, Germany). DNA quantity was measured with the Qubit fluorometer (Life Technologies, Paisley, UK) and amplification performance of the template was evaluated by qPCR. Criteria for processing samples for NGS were ≥ 2 ng/ul DNA amplifiable at Ct ≤ 32 for two different qPCR control assays; based on these criteria, 135 out of 2252 samples (6%) were not processed for NGS due to inadequate DNA.

### NGS and variant analysis

Samples were sequenced in an Ion Proton Sequencer with standard procedures for library and sequencing preparation for a highly-multiplexed previously validated panel [67]. As described, the B-panel that was used in the present series covered ~35 Kb with 373 amplicons in 60 genes, including the entire coding region of TP53, and exons 2, 10, 11, 20 and 21 of PIK3CA. Up to 48 samples were sequenced per PI chip. For data retrieval, base calling was performed on the Torrent Server using Torrent Suite v.3.6.2 and v.4.0. Following base calling and the generation of sequence reads, the pipeline by Torrent Suite included adapter sequence trimming, read alignment to the human reference genome (hg19) and variant calling. Variant annotation was performed by Ion Reporter v.4.0. Raw annotated data from Ion Reporter v.4.0 were evaluated for the reads of all amplicons in the panel and further quality filtered for accepting eligible variants, in the following order: eligible amplicons should have > 100 reads; the variant calling *p*-value threshold was strengthened to 0.0001 instead of the default 0.05; variant position coverage was accepted if > 100; and, variants were accepted for alternative allele coverage > 40; non-annotated variants, as well as indels involving G-stretches (possibly artifacts with semiconductor sequencing) were excluded. Variant allele frequencies of > 5% were accepted by default. The > 100 threshold for amplicon and position coverage was assessed in sample replicates for single nucleotide variants; with this threshold, the Pearson correlation coefficient of variant allele frequencies across replicates was 0.99 (Supplementary File S1, Part B and Figure S1).

For the purposes of the present work, exonic variants in the TP53 and PIK3CA genes were analyzed as mutations if these were non-synonymous and in the case of annotated single nucleotide polymorphisms (SNPs) they had registered minor allele frequency (MAF) < 0.1%, thus excluding even very rare variants in the population [68]. Although methods for bioinformatically predicting the pathogenicity of variants have been succesfully employed in the analysis of germline DNA [69], predicting the functional consequences of cancer mutations has been difficult [70]. Hence we did not attempt to discriminate between driver and passenger mutations using automated methods.

Samples were excluded from the analysis if they had < 10 variants in any of the genes tested and/or if > 90% of the amplicons were covered < 100 times. With this criterion, another 349 samples (15.5%) were considered as technically failed; finally, primary tumors from 1766 patients were eligible for statistical analysis (Figure 6). TP53 and PIK3CA NGS variants were orthogonally validated with dd-sequencing, as previously shown for this panel [67, 71].

### IHC for p53 protein status

IHC was performed on 2um thick TMA sections with the p53 monoclonal antibody (clone DO7; DAKO, Glostrup, DK) at a concentration 1:100 in a Bond MaxTM autostainer (Leica Microsystems, Wetzlar, Germany), upon antigen retrieval in citric acid for 20 min. Tumors with ≥ 10% nuclear staining of any intensity were considered immunopositive for p53 protein [25, 72].

### Statistical analysis

The analysis was conducted in the entire cohort and by local pathology subtyping, separately for pre- (HE10/97 & HE10/00) and post-trastuzumab era studies (HE10/05 & HE10/08).

Continuous variables were presented by the use of various measures (mean, standard deviation, median, range), while categorical variables as frequencies and corresponding percentages. TP53 and PIK3CA mutations were analyzed for the presence or absence as binary variables; as a 4-scale variable for co-mutated tumors; as missense and nonsense/frameshifts; and for domain specificity, according to the most commonly affected coding area for each gene. Associations among demographic, clinical, tumor and treatment characteristics, as well as among p53 protein expression, TP53 and PIK3CA mutations and domains, were examined. For categorical variables the chi-square or Fisher's exact tests were used, where appropriate, while for testing categorical with continuous variables the Mann-Whitney or the Kruskal-Wallis tests were used. Concordance between p53 protein expression and TP53 mutations was assessed by the use of Cohen's Kappa measure of agreement.

The primary endpoint was disease-free survival (DFS), measured from the date of diagnosis until verified disease progression, death or last contact. Only DFS was analyzed for the entire early BC cohort, since follow-up for HE10/08 was still very short. Kaplan-Meier curves and log-rank tests were used for comparing time-to-event distributions, estimated by the product limit method, and evaluating DFS differences. Univariate Cox regression analysis was used for reporting hazard ratios. Univariate Cox with interactions was used for predictive analysis; treatment with trastuzumab was based on the local assessment, thus both TP53 mutations and p53 protein expression were tested for interactions with trastuzumab treatment in centrally assessed HER2-positive patients. Such patients with locally HER2-negative and centrally HER2-positive tumors were not treated with trastuzumab and were therefore used as untreated controls for the predictive analyses. Survival status was updated in June 2014.

For outcome analyses, tumor subtypes classified upon local testing and concordant tumor subtypes, i.e., locally and centrally classified at the same positive/negative status, were examined. Multivariate analysis was conducted by local subtypes (HER2-positive in the pre-trastuzumab era, HER2-positive in the post-trastuzumab era, Luminal A/B, TNBC) in the entire cohort, as well as in the concordant cases. Local subtypes were evaluated because additional treatment (hormone therapy or trastuzumab) was based on this classification. Concordant subtypes were used in order to further validate significant marker effects on outcome.

The models applied for multivariate analyses are shown in Supplementary File S1, Part C. The clinicopathological parameters were chosen by backward elimination among the ones included in each model. The statistically significant interactions from the predictive analysis were also examined upon adjustment for clinicopathological parameters.

All univariate tests were two-sided, with the significance level at α = 0.05. Significance threshold for keeping a variable in the multivariate models was set at α = 0.15, a level higher than usual in order to control for bias in the estimations. Due to the exploratory nature of the study, no correction for multiple testing was made.

The analysis was fully compliant with the reporting recommendations for tumor marker prognostic studies [73]. The SAS software was used for statistical analysis (SAS for Windows, version 9.3, SAS Institute Inc., Cary, NC).

### Patient cohorts

As described above, 1766 patients were eligible for the analysis out of a total of 3491 cases in the four trials; 1585 patients out of 1766 were informative for p53 protein expression. In both cases patients were not selected as representative cases, but based on the availability of tissue samples. Therefore, in order to identify whether the patients eligible for analysis differed from the starting cohorts in each case, with respect to clinicopathological parameters, multivariate logistic regression was used for modeling selection probability. Regarding differences between the 1766 patients and the total cohort (*n* = 3491), the analysis cohort included more patients with high Ki67 labeling, tumor size > 2 cm, lower number of positive nodes, ER/PgR positivity (central assessment) and patients who had received adjuvant radiotherapy (Table S12). Thus, we controlled for differences between selected and original cohorts, by using those parameters as adjustment factors in the multivariate models. The informative cases for p53 protein expression, compared to the analysis cohort, comprised of more patients with high values in CEN17 and Ki67, as well as with histological grade III (Table S13).

## SUPPLEMENTARY MATERIAL FIGURES AND TABLES








