# Supplementary material for: TP53 mutations and protein immunopositivity may predict for poor outcome but also for trastuzumab benefit in patients with early breast cancer treated in the adjuvant setting
Source: Oncotarget. 2016 Apr 26;7(22):32731–53. doi: 10.18632/oncotarget.9022 (PMC5078047; doi:10.18632/oncotarget.9022)
Supplement: Supplementary file 1 [file oncotarget-07-32731-s001.pdf]

TP53 mutations and protein expression may predict for poor outcome but also for trastuzumab benefit in patients with early breast cancer treated in the adjuvant setting

Supplementary Materials

Supplementary File S1 Part A

PART A: Comparison between HER2-positive cohorts

		HER2 pos, T	HER2 pos, no T	Chi-square *p*-value	HER2 centrally & locally positive	HER2 centrally positive & locally negative	Chi-square *p*-value
**ERPgR local**	**Negative**	101 (36.5)	58 (28.9)	0,081	79 (44.6)	7 (14.3)	< 0.001
	**Positive**	176 (63.5)	143 (71.1)		98 (55.4)	42 (85.7)	
**ERPgR central**	**Negative**	83 (31.0)	60 (32.3)	0,77	67 (37.8)	11 (22.0)	0,037
	**Positive**	185 (69.0)	126 (67.7)		110 (62.2)	39 (78.0)	

Supplementary File S1 Part B

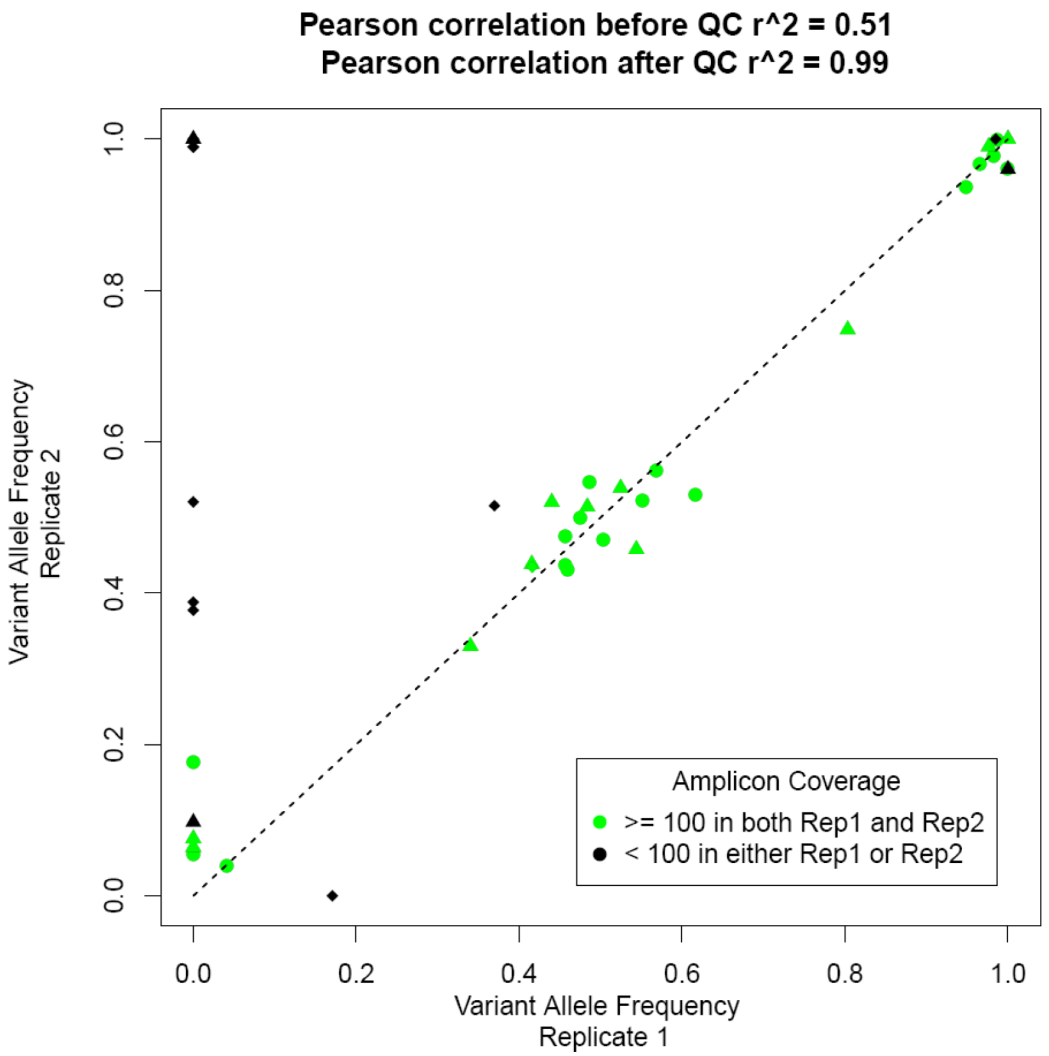

Supplementary Figure S1: Justification of the 100-coverage threshold for accepting sequence variants with the applied panel. In order to establish a threshold for accepting the individual variants that were returned by the Ion Reporter platform v.4, we used results from 3 FFPE DNA replicates. We evaluated the concordance of variant allele frequencies between replicates by calculating the Pearson correlation coefficient before and after Quality Control (QC). Variants that were not found in one of the two replicates were assigned zero frequency. As shown, the initial correlation between variant frequencies was poor (r^2 = 0.51). By filtering variants with amplicon coverage less than 100, the correlation coefficient between the frequencies was 0.99 across all replicates. Circles, triangles and diamonds represent frequencies from each individual pair of replicate samples. Green: variant allele frequencies of variants that remain after QC (corresponding to amplicons with read coverage ≥ 100); black: variant allele frequencies of variants that are filtered out after QC (variants with amplicon coverage < 100 times).

Supplementary File S1 Part C

Factors (parameters) in Multivariate models

Forest plot (Figure 5):

Luminal A/B

1. Menopausal status (post vs. pre)
2. Number of positive nodes (≥ 4 vs. 0–3)
3. Histological type (invasive ductal vs. lobular vs. mixed vs. other)
4. Histological grade (I vs. II vs. III)
5. Adjuvant hormonotherapy (Yes vs. No)
6. Adjuvant radiotherapy (Yes vs. No)
7. Tumor size (> 2 cm vs. ≤ 2 cm)
8. Ki67 (continuous: incr. by 5%)

TNBC

1. Menopausal status (post vs. pre)
2. Number of positive nodes (≥ 4 vs. 0–3)
3. Histological type (invasive ductal vs. lobular vs. mixed vs. other)
3. Histological grade (I–II vs. III)
4. Adjuvant radiotherapy (Yes vs. No)
5. Tumor size (> 2 cm vs. ≤ 2 cm)
6. Ki67 (continuous: incr. by 5%)

HER2pos, pre T

1. Menopausal status (post vs. pre)
2. Number of positive nodes (≥ 4 vs. 0–3)
3. Histological type (invasive ductal vs. lobular vs. mixed vs. other)
4. Histological grade (I–II vs. III)
5. Adjuvant hormonotherapy (Yes vs. No)
6. Adjuvant radiotherapy (Yes vs. No)
7. Tumor size (> 2 cm vs. ≤ 2 cm)
8. Ki67 (continuous: incr. by 5%)

HER2pos, post T

1. Menopausal status (post vs. pre)
2. Number of positive nodes (≥ 4 vs. 0–3)
3. Histological type (invasive ductal vs. lobular vs. mixed vs. other)
4. Histological grade (I–II vs. III)
5. Adjuvant hormonotherapy (Yes vs. No)
6. Adjuvant radiotherapy (Yes vs. No)
7. Tumor size (> 2 cm vs. ≤ 2 cm)
8. Ki67 (continuous: incr. by 5%)

Table S5:

1. Menopausal status (post vs. pre)
2. Number of positive nodes (≥ 4 vs. 0–3)
3. Histological type (invasive ductal vs. lobular vs. mixed vs. other)
4. Histological grade (I–II vs. III)
5. Adjuvant hormonotherapy (Yes vs. No)
6. Adjuvant radiotherapy (Yes vs. No)
7. Tumor size (> 2 cm vs. ≤ 2 cm)
8. Ki67 (continuous: incr. by 5%)

Table S6:

Luminal A/B

1. Menopausal status (post vs. pre)
2. Number of positive nodes (≥ 4 vs. 0–3)
3. Histological type (invasive ductal vs. lobular vs. mixed vs. other)
4. Histological grade (I vs. II vs. III)
5. Adjuvant hormonotherapy (Yes vs. No)
6. Adjuvant radiotherapy (Yes vs. No)
7. Tumor size (> 2 cm vs. ≤ 2 cm)
8. Ki67 (continuous: incr. by 5%)

TNBC

1. Menopausal status (post vs. pre)
2. Number of positive nodes (≥ 4 vs. 0–3)
3. Histological type (invasive ductal vs. lobular vs. mixed vs. other)
4. Histological grade (I –II vs. III)
5. Adjuvant radiotherapy (Yes vs. No)
6. Tumor size (> 2 cm vs. ≤ 2 cm)
7. Ki67 (continuous: incr. by 5%)

HER2pos, pre T

1. Menopausal status (post vs. pre)
2. Number of positive nodes (≥ 4 vs. 0–3)
3. Histological type (invasive ductal vs. lobular vs. mixed vs. other)
4. Histological grade (I–II vs. III)
5. Adjuvant hormonotherapy (Yes vs. No)
6. Adjuvant radiotherapy (Yes vs. No)
7. Tumor size (> 2 cm vs. ≤ 2 cm)
8. Ki67 (continuous: incr. by 5%)

HER2pos, post T

1. Menopausal status (post vs. pre)
2. Number of positive nodes (≥4 vs. 0–3)
3. Histological type (invasive ductal vs. lobular vs. mixed vs. other)
4. Histological grade (I–II vs. III)
5. Adjuvant hormonotherapy (Yes vs. No)

6. Adjuvant radiotherapy (Yes vs. No)
7. Tumor size (> 2 cm vs. ≤ 2 cm)
8. Ki67 (continuous: incr. by 5%)

Table S8:

Luminal A/B

9. Menopausal status (post vs. pre)
10. Number of positive nodes (≥ 4 vs. 0–3)
11. Histological type (invasive ductal vs. lobular vs. mixed vs. other)
12. Histological grade (I vs. II vs. III)
13. Adjuvant hormonotherapy (Yes vs. No)
14. Adjuvant radiotherapy (Yes vs. No)
15. Tumor size (> 2 cm vs. ≤ 2 cm)
16. Ki67 (continuous: incr. by 5%)

TNBC

8. Menopausal status (post vs. pre)
9. Number of positive nodes (≥4 vs. 0–3)
10. Histological type (invasive ductal vs. lobular vs. mixed vs. other)
11. Histological grade (I –II vs. III)
12. Adjuvant radiotherapy (Yes vs. No)
13. Tumor size (> 2 cm vs. ≤ 2 cm)
14. Ki67 (continuous: incr. by 5%)

HER2pos, pre T

9. Menopausal status (post vs. pre)
10. Number of positive nodes (≥4 vs. 0–3)
11. Histological type (invasive ductal vs. lobular vs. mixed vs. other)
12. Histological grade (I–II vs. III)
13. Adjuvant hormonotherapy (Yes vs. No)
14. Adjuvant radiotherapy (Yes vs. No)
15. Tumor size (> 2 cm vs. ≤ 2 cm)
16. Ki67 (continuous: incr. by 5%)

HER2pos, post T

9. Menopausal status (post vs. pre)
10. Number of positive nodes (≥ 4 vs. 0–3)
11. Histological type (invasive ductal vs. lobular vs. mixed vs. other)
12. Histological grade (I–II vs. III)
13. Adjuvant hormonotherapy (Yes vs. No)
14. Adjuvant radiotherapy (Yes vs. No)
15. Tumor size (> 2 cm vs. ≤ 2 cm)
16. Ki67 (continuous: incr. by 5%)

Table S9:

Luminal A/B

17. Menopausal status (post vs. pre)
18. Number of positive nodes (≥ 4 vs. 0–3)
19. Histological type (invasive ductal vs. lobular vs. mixed vs. other)
20. Histological grade (I vs. II vs. III)
21. Adjuvant hormonotherapy (Yes vs. No)
22. Adjuvant radiotherapy (Yes vs. No)
23. Tumor size (> 2 cm vs. ≤ 2 cm)
24. Ki67 (continuous: incr. by 5%)

TNBC

15. Menopausal status (post vs. pre)
16. Number of positive nodes (≥ 4 vs. 0–3)
17. Histological type (invasive ductal vs. lobular vs. mixed vs. other)
18. Histological grade (I –II vs. III)
19. Adjuvant radiotherapy (Yes vs. No)
20. Tumor size (>2 cm vs. ≤ 2 cm)
21. Ki67 (continuous: incr. by 5%)

HER2pos, pre T

17. Menopausal status (post vs. pre)
18. Number of positive nodes (≥ 4 vs. 0–3)
19. Histological type (invasive ductal vs. lobular vs. mixed vs. other)
20. Histological grade (I–II vs. III)
21. Adjuvant hormonotherapy (Yes vs. No)
22. Adjuvant radiotherapy (Yes vs. No)
23. Tumor size (> 2 cm vs. ≤ 2 cm)
24. Ki67 (continuous: incr. by 5%)

HER2pos, post T

17. Menopausal status (post vs. pre)
18. Number of positive nodes (≥ 4 vs. 0–3)
19. Histological type (invasive ductal vs. lobular vs. mixed vs. other)
20. Histological grade (I–II vs. III)
21. Adjuvant hormonotherapy (Yes vs. No)
22. Adjuvant radiotherapy (Yes vs. No)
23. Tumor size (> 2 cm vs. ≤ 2 cm)
24. Ki67 (continuous: incr. by 5%)

Supplementary Table S1: Associations of TP53 and PIK3CA mutations with clinicopathological variables

Supplementary Table S2: Associations of p53 IHC (10% cut-off) with clinicopathological variables

	p53 IHC (positive if ≥ 10%)		
	**Negative**	**Positive**	***p*-value**
**Patients**			
N	737	848	
**Age (years)**			
Median	53.5	52.5	0.066
Min-Max	(22.4–79.3)	(21.0–82.9)	
**Tumor size**			
Median	2.5	2.5	0.13
Min-Max	(0.0–11.5)	(0.0–14.8)	
**Ki67**			
Median	20	25	< 0.001
Min-Max	(0.0–100.0)	(0.0–100.0)	
**CEN17 copies**			
Median	2	2.1	0.51
Min-Max	(0.6–15.2)	(1.0–17.5)	
	***N* (%)**	***N* (%)**	
**Age**			
≤ 50	276 (37.5)	374 (44.0)	0.009
> 50	460 (62.5)	476 (56.0)	
**Menopausal status**			
Postmenopausal	414 (56.3)	435 (51.2)	0.043
Premenopausal	322 (43.8)	415 (48.8)	
**Tumor size**			
≤ 2	245 (33.3)	323 (38.0)	0.051
> 2	491 (66.7)	527 (62.0)	
**Positive lymph nodes**			
0–3	441 (59.9)	517 (60.8)	0.71
≥ 4	295 (40.1)	333 (39.2)	
**Histological grade**			
I	49 (6.7)	51 (6.0)	0.19
II	337 (45.9)	355 (42.0)	
III	348 (47.4)	440 (52.0)	
**Histological type**			
DIC-NST	588 (79.9)	714 (84.0)	0.11
Invasive lobular	70 (9.5)	65 (7.6)	
Mixed	35 (4.8)	39 (4.6)	
Other	43 (5.8)	32 (3.8)	
**Surgery (binary)**			
MRM	439 (59.6)	459 (54.1)	0.027

Other	297 (40.4)	389 (45.9)	
**Hormonotherapy**			
No	168 (22.9)	200 (23.6)	0.71
Yes	567 (77.1)	646 (76.4)	
**Radiotherapy**			
No	181 (25.3)	178 (21.5)	0.079
Yes	534 (74.7)	649 (78.5)	
**Subtypes, entire cohort**			
Luminal A	258 (35.0)	239 (28.2)	0.002
Luminal B	198 (26.9)	245 (28.9)	
Luminal-HER2	129 (17.5)	151 (17.8)	
HER2-Enriched	50 (6.8)	100 (11.8)	
TNBC	102 (13.8)	113 (13.3)	
**Subtypes, entire cohort, combined**			
Luminal A/B	456 (61.9)	484 (57.1)	0.058
HER2-positive	179 (24.3)	251 (29.6)	
TNBC	102 (13.8)	113 (13.3)	
**Subtypes concordant***			
Luminal A	35 (6.4)	53 (8.7)	0.13
Luminal B	226 (41.5)	221 (36.2)	
Luminal-HER2	167 (30.7)	206 (33.7)	
HER2-Enriched	60 (11.0)	80 (13.1)	
TNBC	56 (10.3)	51 (8.3)	
**Subtype concordant combined**			
Luminal A/B	393 (72.2)	426 (70.0)	0.14
HER2-positive	95 (17.5)	132 (21.7)	
TNBC	56 (10.3)	51 (8.4)	
**Basal**			
Basal	144 (19.7)	205 (24.3)	0.029
non-Basal	587 (80.3)	639 (75.7)	
**RandomGroup**			
E-CMF	20 (2.7)	61 (7.2)	< 0.001
E-CMF-Doc	65 (8.8)	99 (11.6)	
E-CMF-T	69 (9.4)	105 (12.4)	
E-T-CMF	472 (64.0)	477 (56.1)	
ET-CMF	111 (15.1)	108 (12.7)	

Notes: MRM: modified radical mastectomy; *: same ER/PgR and HER2 status upon local and central testing.

Supplementary Table S3: Associations between mutations and p53 IHC, including concordance evaluation between TP53 mutations and IHC

	p53 IHC 10% cut off								
	< 10%			≥ 10%			*Total*	Cohen's Kappa	Chi-square *p*-value
	N	row %	col %	*N*	row %	col %			
**TP53_MUT**									
**NO**	647	52.4	87.8	587	47.6	69.2	*1234*	0.18 (0.14–0.22)	< 0.001
**YES**	90	25.6	12.2	261	74.4	30.8	*351*		
Total	*737*			*848*			*1585*		
**PIK3CA_MUT**									
**NO**	556	48.0	75.4	602	52.0	71.0	*1158*		0.046
**YES**	181	42.4	24.6	246	57.6	29.0	*427*		
Total	*737*			848			*1585*		

Supplementary Table S4: Univariate cox testing for interaction between mutation parameters and Luminal A vs. Luminal B subtypes

Mutation parameter	comparison	*N* patients	*N* events	HR	95%CI	Wald's *p*
**TP53 MUT**	TP53 MUT YES vs NO At LumA	49 vs. 539	16 vs. 87	2.24	1.31–3.81	0.28
	TP53 MUT YES vs NO At LumB	94 vs. 369	31 vs. 84	1.54	1.02–2.33	
	LumB vs LumA At TP53 MUT = NO	369 vs. 539	84 vs. 87	1.34	0.99–1.81	
	LumB vs LumA At TP53 MUT = YES	94 vs. 49	31 vs. 16	0.93	0.51–1.70	
**TP53 domains**	TP53 domains DBD vs none At LumA	31 vs. 539	7 vs. 87	1.41	0.65–3.04	*0.043*
	TP53 domains other vs none At LumA	18 vs. 539	9 vs. 87	4.14	2.08–8.22	
	TP53 domains DBD vs none At LumB	62 vs. 369	23 vs. 84	1.71	1.08–2.72	
	TP53 domains other vs none At LumB	32 vs. 369	8 vs. 84	1.20	0.58–2.49	
	LumB vs LumA At TP53 domains = none	369 vs. 539	84 vs. 87	1.34	0.99–1.81	
	LumB vs LumA At TP53 domains = DBD	62 vs. 31	23 vs. 7	1.63	0.70–3.81	
	LumB vs LumA At TP53 domains = other	32 vs. 18	8 vs. 9	0.39	0.15–1.01	
**PIK3CA MUT**	PIK3CA MUT YES vs NO At LumA	195 vs. 393	30 vs. 73	0.79	0.52–1.21	0.44
	PIK3CA MUT YES vs NO At LumB	136 vs. 327	34 vs. 81	1.00	0.67–1.49	
	LumB vs LumA At PIK3CA MUT = NO	327 vs. 393	81 vs. 73	1.26	0.92–1.73	
	LumB vs LumA At PIK3CA MUT = YES	136 vs. 195	34 vs. 30	1.59	0.97–2.60	
**PIK3CA domains**	PIK3CA domains KINASE vs none At LumA	94 vs. 393	14 vs. 73	0.75	0.42–1.33	0.54
	PIK3CA domains other vs none At LumA	101 vs. 393	16 vs. 73	0.84	0.49–1.44	
	PIK3CA domains KINASE vs none At LumB	91 vs. 327	24 vs. 81	1.12	0.71–1.76	
	PIK3CA domains other vs none At LumB	45 vs. 327	10 vs. 81	0.80	0.41–1.54	
	LumB vs LumA At PIK3CA domains = none	327 vs. 393	81 vs. 73	1.26	0.92–1.73	
	LumB vs LumA At PIK3CA domains = KINASE	91 vs. 94	24 vs. 14	1.88	0.97–3.63	
	LumB vs LumA At PIK3CA domains = other	45 vs. 101	10 vs. 16	1.20	0.54–2.65	
**TP53 or PIK3CA MUT**	TP53 or PIK3CA MUT YES vs NO At LumA	225 vs. 363	39 vs. 64	0.96	0.64–1.42	0.47
	TP53 or PIK3CA MUT YES vs NO At LumB	198 vs. 265	53 vs. 62	1.17	0.81–1.69	
	LumB vs LumA At TP53 or PIK3CA MUT = NO	265 vs. 363	62 vs. 64	1.24	0.88–1.77	
	LumB vs LumA At TP53 or PIK3CA MUT = YES	198 vs. 225	53 vs. 39	1.52	1.00–2.30	
**MUT breakdown**	PIK3CA only vs NONE At LumA	176 vs. 363	23 vs. 64	0.70	0.44–1.13	0.67
	TP53 only vs NONE At LumA	30 vs. 363	9 vs. 64	1.79	0.89–3.60	
	both vs NONE At LumA	19 vs. 363	7 vs. 64	2.38	1.09–5.20	
	PIK3CA only vs NONE At LumB	104 vs. 265	22 vs. 62	0.89	0.55–1.45	
	TP53 only vs NONE At LumB	62 vs. 265	19 vs. 62	1.39	0.83–2.33	
	both vs NONE At LumB	32 vs. 265	12 vs. 62	1.70	0.91–3.16	
	LumB vs LumA At = NONE	265 vs. 363	62 vs. 64	1.24	0.88–1.77	
	LumB vs LumA At = PIK3CA only	104 vs. 176	22 vs. 23	1.58	0.88–2.84	
	LumB vs LumA At = TP53 only	62 vs. 30	19 vs. 9	0.96	0.44–2.14	
	LumB vs LumA At = both	32 vs. 19	12 vs. 7	0.89	0.35–2.26	

Supplementary Table S5: Univariate Cox analysis for clinicopathological parameters in the entire cohort against disease-free survival

Supplementary Table S6: Multivariable analysis in centrally assessed HER2-positive tumors, including interactions between trastuzumab and TP53-related parameters

Parameter	*N* patients	*N* events	HR	95% CI	Wald's *p*
**HER2 positive, post-trastuzumab, *N* = 208; p53 IHC interaction included in the model**					
Histological grade III vs. I–II	137 vs. 71	15 vs. 14	0.54	0.26–1.13	0.10
Radiotherapy Yes vs. No	158 vs. 50	25 vs. 4	2.26	0.77–6.64	0.14
p53 IHC 10% cut off * T-treatment					0.015
p53 IHC ≥ 10% vs < 10% @ T-treated	112 vs. 51	5 vs. 13	0.16	0.06–0.44	
p53 IHC ≥ 10% vs < 10% @ non-T-treated	25 vs. 20	6 vs. 5	1.09	0.33–3.60	
T-treated vs non-T-treated @ p53 IHC < 10%	51 vs. 20	13 vs. 5	0.98	0.35–2.77	
T-treated vs non-T-treated @ p53 IHC ≥ 10%	112 vs. 25	5 vs. 6	0.14	0.04–0.46	
**HER2 positive, post-trastuzumab, *N* = 224; TP53 MUT interaction included in the model**					
Histological type, Invasive lobular vs. ductal	4 vs. 210	3 vs. 26	5.35	1.48–19.31	0.010
Histological type, mixed vs. ductal	3 vs. 210	1 vs. 26	2.30	0.30–17.67	0.42
Histological type, other vs. ductal	7 vs. 210	1 vs. 26	0.59	0.07–4.69	0.62
Radiotherapy Yes vs. No	169 vs. 55	27 vs. 4	2.21	0.77–6.37	0.14
TP53 MUT YES/NO * T-treatment					0.010
TP53_MUT YES vs NO @ T-treated	75 vs. 100	4 vs. 15	0.37	0.12–1.11	
TP53_MUT YES vs NO @ non-T-treated	13 vs. 36	5 vs. 7	3.17	0.93–10.80	
T-treated vs non-T-treated @ TP53_MUT, NO	100 vs. 36	15 vs. 7	0.78	0.30–2.02	
T-treated vs non-T-treated @ TP53_MUT, YES	75 vs. 13	4 vs. 5	0.09	0.02–0.35	

Notes: T: trastuzumab; IHC: Immunohistochemistry; *: interaction.

Supplementary Table S7: Multivariable analysis in concordant cases only (total *N* = 1248) by using the same adjustment as in the entire cohort

	N patients	*N* events	HR	95% CI	Wald's *p*
**HER2-positive, no T (HE10/97 and HE10/00) (*N* = 88)**					
Nodes positive categorical ≥ 4 vs. 0–3	48 vs. 40	23 vs. 6	3.94	1.60–9.68	0.003
Tumor size categorical > 2 vs. ≤ 2 cm	68 vs. 20	26 vs. 3	2.88	0.87–9.51	0.083
**HER2-positive, T-treated (HE10/05 and HE10/08) (*N* = 142)**					
Nodes positive categorical ≥ 4 vs. 0-3	52 vs. 90	12 vs. 8	2.24	0.90–5.53	0.082
p53 IHC ≥ 10% vs. < 10%	87 vs. 55	6 vs. 14	0.28	0.11–0.73	0.010
**Luminal A/B (*N* = 888)**					
Nodes positive categorical ≥ 4 vs. 0–3	355 vs. 533	117 vs. 54	2.95	2.12–4.10	< 0.001
Histological grade					*0.12*
Histological grade II vs. I	491 vs. 84	88 vs. 7	1.48	0.68–3.22	0.32
Histological grade III vs. I	313 vs. 84	76 vs. 7	1.91	0.87–4.19	0.10
Tumor size categorical > 2 vs. ≤ 2 cm	549 vs. 339	130 vs. 41	1.54	1.08–2.19	0.018
TP53 mutations YES vs. NO	112 vs. 776	35 vs. 136	1.69	1.16–2.47	0.006
**TNBC (*N* = 111)**					
Nodes positive categorical ≥ 4 vs. 0–3	39 vs. 72	17 vs. 7	4.85	2.00–11.78	< 0.001

Notes: T: trastuzumab; IHC: Immunohistochemistry ; Luminal A/B: ER/PgR positive, HER2-negative.

Supplementary Table S8: Tumor typing according to local and central testing

**ER/PgR status**	***N***	**%**
Locally positive / centrally positive	1462	86.4
Locally negative / centrally positive	150	8.9
Locally positive / centrally negative	81	4.8
Total Assessed cases	1693	
Total Discordant cases	231	13.6
**HER2 status**	***N***	%
Locally positive / centrally positive	1462	87.5
Locally negative / centrally positive	74	4.4
Locally positive / centrally negative	135	8.1
Total Assessed cases	1671	
Total Discordant cases	209	12.5

Supplementary Table S9: Multivariable analysis (DFS) for mutant gene domains in the entire cohort

	N patients	*N* events	HR	95% CI	Wald's *p*
**HER2-positive, no T (HE10/97 & HE10/00) (*N* = 178)**					
Nodes positive categorical ≥ 4 vs. 0–3	96 vs. 82	44 vs. 17	2.31	1.31–4.10	0.004
Tumor size categorical > 2 vs. ≤ 2 cm	138 vs. 40	54 vs. 7	2.61	1.18–5.77	0.017
p53 IHC ≥ 10% vs. < 10%	92 vs. 86	38 vs. 23	1.56	0.92–2.65	0.098
**HER2-positive, T-treated (HE10/05 & HE10/08) (*N* = 252)**					
Nodes positive categorical ≥ 4 vs. 0–3	79 vs. 173	160 vs. 92	2.09	1.02–4.26	0.043
p53 IHC ≥ 10% vs. < 10%	17 vs. 15	12 vs. 20	0.37	0.18–0.77	0.008
**Luminal A/B (*N* = 1044)**					
Nodes positive categorical ≥ 4 vs. 0–3	422 vs. 622	141 vs. 76	2.46	1.85–3.26	< 0.001
Histological grade					0.025
Histological grade II vs. I	558 vs. 98	109 vs. 8	1.95	0.95–4.01	0.069
Histological grade III vs. I	388 vs. 98	100 vs. 8	2.47	1.20–5.10	0.015
Tumor size categorical > 2 vs. ≤ 2 cm	653 vs. 391	164 vs. 53	1.54	1.13–2.11	0.007
TP53 mutant domains					0.011
DBD vs. none	93 vs. 902	30 vs. 170	1.65	1.11–2.45	0.013
Other vs. none	49 vs. 902	17 vs. 170	1.68	1.01–2.77	0.044
**TNBC (*N* = 235)**					
Nodes positive categorical ≥ 4 vs. 0–3	82 vs. 153	33 vs. 26	2.41	1.43–4.04	0.001
Histological grade III vs. I–II	180 vs. 55	38 vs. 21	0.47	0.27–0.82	0.008
TP53 domains					0.060
DBD vs. none	41 vs. 150	15 vs. 32	2.11	1.12–3.97	0.021
Other vs. none	44 vs. 150	12 vs. 32	1.53	0.78–3.01	0.22

Notes: T: trastuzumab; IHC: Immunohistochemistry; DBD: DNA binding domain; Luminal A/B: ER/PgR positive, HER2 negative.

Supplementary Table S10: Multivariable analysis (DFS) for mutant gene domains in concordant cases only

	***N* patients**	***N* events**	**HR**	**95% CI**	**Wald's *p***
**HER2-positive, no T (HE10/97 & HE10/00) (*N* = 88)**					
Nodes positive categorical ≥ 4 vs. 0–3	48 vs. 40	23 vs. 6	3.94	1.60–9.68	0.003
Tumor size categorical > 2 vs. ≤ 2 cm	68 vs. 20	26 vs. 3	2.88	0.87–9.51	0.083
**HER2-positive, T-treated (HE10/05 & HE10/08) (*N* = 142)**					
Nodes positive categorical ≥ 4 vs. 0–3	52 vs. 90	12 vs. 8	2.24	0.90–5.53	0.082
p53 IHC ≥ 10% vs. < 10%	87 vs. 55	6 vs. 14	0.28	0.11–0.73	0.010
**Luminal A/B (*N* = 888)**					
Nodes positive categorical ≥ 4 vs. 0–3	355 vs. 533	117 vs. 54	2.95	2.12-4.10	< 0.001
Histological grade					0.12
Histological grade II vs. I	491 vs. 84	88 vs. 7	1.48	0.68–3.22	0.32
Histological grade III vs. I	313 vs. 84	76 vs. 7	1.91	0.87–4.19	0.10
Tumor size categorical > 2 vs. ≤ 2 cm	549 vs. 339	130 vs. 41	1.53	1.07–2.19	0.019
TP53 mutant domains					0.023
DBD vs. none	71 vs. 776	21 vs. 136	1.67	1.05–2.66	0.031
other vs. none	41 vs. 776	14 vs. 136	1.73	0.99–3.01	0.053
**TNBC (N = 111)**					
Nodes positive categorical ≥ 4 vs. 0–3	39 vs. 72	17 vs. 7	4.85	2.00–11.78	< 0.001

Notes: T: trastuzumab; IHC: Immunohistochemistry; DBD: DNA binding domain; Luminal A/B: ER/PgR-positive, HER2-negative.

Supplementary Table S11: Description of the 4 HeCOG trials for patients with operable high-risk breast cancer

Supplementary Table S12: Comparison between the entire cohort of 3491 patients who participated in the four adjuvant trials vs. the study cohort of 1766 patients with informative NGS data

Odds ratio estimates			
**Effect**	**OR**	**95% CI**	**Wald's *p***
**CEN17 copies (incr. by 1)**			
	0.99	0.93–1.06	0.75
**Ki67 (% of positive cells, incr. by 5)**			
	1.02	1.00–1.04	0.034
**Age (years)**			
> 50 vs. ≤ 50	1.23	0.88–1.74	0.23
**Menopausal status**			
Post vs. Pre	0.80	0.57–1.11	0.18
**Tumor size in cm**			
> 2 vs. ≤ 2	1.22	1.00–1.48	0.055
**Positive lymph nodes (number)**			
≥ 4 vs. 0–3	0.58	0.46–0.72	< 0.001
**Histological grade**			
III vs. I–II	1.04	0.85–1.28	0.72
**Histological type**			0.13
Invasive lobular vs. Invasive ductal	0.70	0.52–0.96	0.12
Mixed vs. Invasive ductal	0.81	0.54–1.20	0.66
Other vs. Invasive ductal	1.00	0.63–1.59	0.45
**Surgery (binary)**			
Other vs. MRM	1.16	0.93–1.46	0.19
**Hormonotherapy**			
Yes vs. No	0.75	0.52–1.10	0.14
**Radiotherapy**			
Yes vs. No	1.37	1.05–1.78	0.021
**ER/PgR central**			
Positive vs. Negative	1.47	1.08–2.01	0.016
**ER/PgR local**			
Positive vs. Negative	1.09	0.73–1.63	0.67
**EGFR central**			
Positive vs. Negative	1.29	0.93–1.78	0.13
**CK5 central**			
Positive vs. Negative	0.92	0.63–1.33	0.64
**HER2 IHC local**			
Overexpression vs. no overexpression	0.81	0.61–1.07	0.13
**HER2 status central**			
Positive vs. Negative	1.29	0.95–1.75	0.11

Notes: MRM: modified radical mastectomy; IHC: Immunohistochemistry.

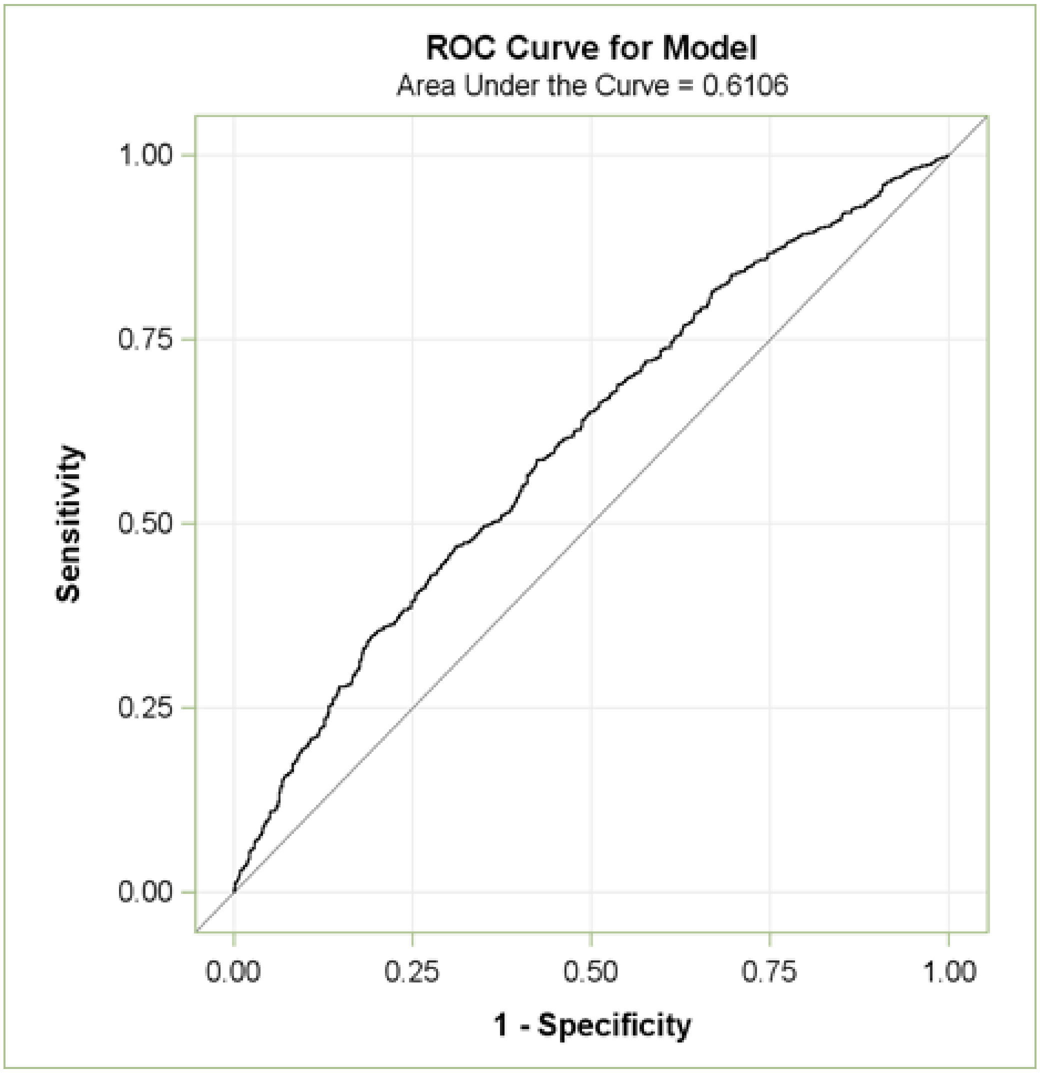
ROC Curve for Model
Area Under the Curve = 0.6106
Sensitivity
1.00
0.75
0.50
0.25
0.00
0.00
0.25
0.50
0.75
1.00
1 - Specificity

Supplementary Table S13: Comparison between the 1766 patients with informative NGS data vs. the 1585 patients with informative p53 IHC

Odds ratio estimates			
**Effect**	**OR**	**95% CI**	**Wald's *p***
**CEN17 copies (incr. by 1)**			
	1.39	1.02–1.90	0.037
**Ki67 (% of positive cells, incr. by 5)**			
	1.09	1.02–1.16	0.010
**Age (years)**			
> 50 vs. ≤ 50	1.37	0.58–3.22	0.47
**Menopausal status**			
Post vs. Pre	0.65	0.28–1.52	0.32
**Tumor size in cm**			
> 2 vs. ≤ 2	1.13	0.68–1.89	0.63
**Positive lymph nodes**			
≥ 4 vs. 0–3	0.66	0.37–1.18	0.16
**Histological grade**			
III vs. I–II	1.77	1.02–3.07	0.041
**Histological type**			0.40
Invasive lobular vs. Invasive ductal	1.49	0.65–3.45	0.90
Mixed vs. Invasive ductal	0.75	0.31–1.84	0.14
Other vs. Invasive ductal	3.60	0.48–26.9	0.23
**Surgery (binary)**			
Other vs. MRM	1.35	0.74–2.47	0.32
**Hormonotherapy**			
Yes vs. No	1.05	0.40–2.77	0.93
**Radiotherapy**			
Yes vs. No	1.44	0.74–2.79	0.29
**ER/PgR central**			
Positive vs. Negative	1.21	0.51–2.87	0.67
**ER/PgR local**			
Positive vs. Negative	1.02	0.35–3.00	0.97
**EGFR central**			
Positive vs. Negative	0.80	0.35–1.85	0.60
**CK5 central**			
Positive vs. Negative	1.76	0.57–5.47	0.33
**HER2 IHC local**			
Overexpression vs. no overexpression	0.82	0.40–1.65	0.57
**HER2 status central**			
Positive vs. Negative	1.30	0.58–2.93	0.52

Notes: MRM: modified radical mastectomy; IHC: Immunohistochemistry.

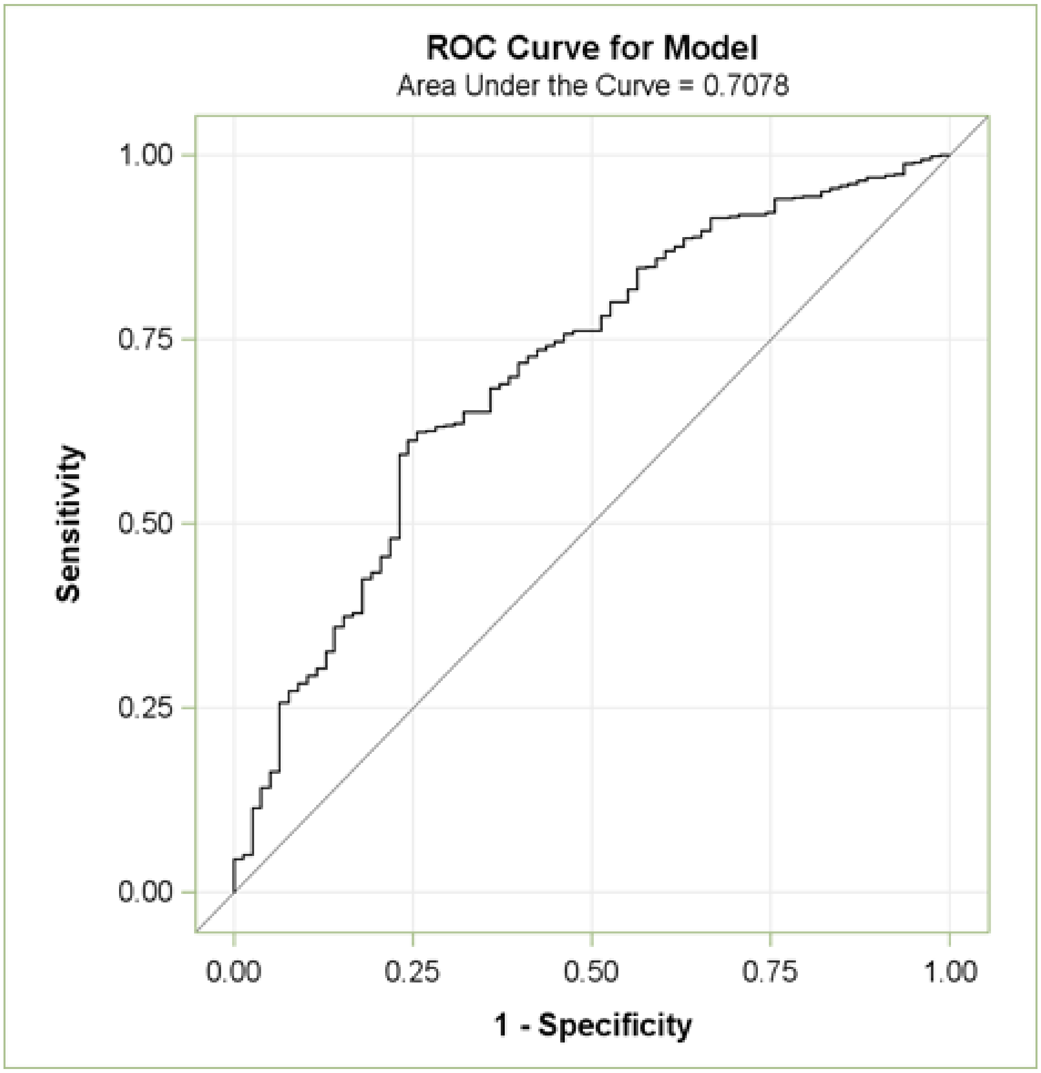
ROC Curve for Model
Area Under the Curve = 0.7078
Sensitivity
1.00
0.75
0.50
0.25
0.00
0.00
0.25
0.50
0.75
1.00
1 - Specificity
