# Supplementary material for: TP53 mutations and protein immunopositivity may predict for poor outcome but also for trastuzumab benefit in patients with early breast cancer treated in the adjuvant setting
Source: Oncotarget. 2016 Apr 26;7(22):32731–53. doi: 10.18632/oncotarget.9022 (PMC5078047; doi:10.18632/oncotarget.9022)
Supplement: Supplementary file 2 [file oncotarget-07-32731-s002.pdf]

Table S1: Associations of TP53 and PIK3CA mutations with clinicopathological variables.

	TP53 MUTATIONS			TP53 MUT DOMAINS					PIK3CA MUTATIONS			PIK3CA MUT DOMAINS			
	NO	YES	p-value	NONE	TAD	DBD	TETRA	p-value	NO	YES	p-value	NONE	HELICAL	KINASE	p-value
**Patients**															
N	1.386	380		1.386	81	225	74		1.308	458		1.308	191	265	
**Age (years)**															
Median	52,9	53,1	0,39	52,9	53,5	52,4	55,6	0,58	52,9	53	0,69	52,9	51,9	54,1	0,66
Min-Max	(22.4-82.9)	(21.0-76.8)		(22.4-82.9)	(30.6-76.7)	(21.0-76.8)	(25.4-75.0)		(23.8-82.9)	(21.0-77.7)		(21.0-82.9)	(31.3-75.7)	(22.4-77.7)	
**Tumor size**															
Median	2,5	2,5	0,064	2,5	2,9	2,6	2,4	0,14	2,5	2,5	0,74	2,5	2,5	2,5	0,81
Min-Max	(0.0-14.8)	(0.0-12.0)		(0.0-14.8)	(0.8-12.0)	(0.5-10.5)	(0.0-8.0)		(0.0-14.8)	(0.0-11.5)		(0.0-14.8)	(0.0-9.5)	(0.0-11.5)	
**Ki67**															
Median	18	35	<0.001	18	39	35	37,5	<0.001	25	16	<0.001	25	13	20	<0.001
Min-Max	(0.0-100.0)	(0.0-100.0)		(0.0-100.0)	(1.0-100.0)	(0.0-100.0)	(0.0-98.0)		(0.0-100.0)	(0.0-95.0)		(0.0-100.0)	(0.0-85.0)	(0.0-95.0)	
**CEN17 copies**															
Median	2	2,2	<0.001	2	2,4	2,2	2,2	0,005	2	2	0,40	2	2	2,1	0,60
Min-Max	(0.7-17.5)	(0.6-13.8)		(0.7-17.5)	(1.0-10.3)	(1.0-13.8)	(0.6-5.2)		(0.6-17.5)	(1.0-11.4)		(0.6-17.5)	(1.0-11.4)	(1.0-7.7)	
	N (%)	N (%)		N (%)	N (%)	N (%)	N (%)		N (%)	N (%)		N (%)	N (%)	N (%)	
**Age**															
≤50	572 (41.3)	152 (40.2)	0,71	572 (41.3)	34 (42.5)	95 (42.2)	23 (31.5)	0,40	539 (41.3)	185 (40.4)	0,74	539 (41.3)	79 (41.4)	105 (39.6)	0,88
>50	814 (58.7)	226 (59.8)		814 (58.7)	46 (57.5)	130 (57.8)	50 (68.5)		767 (58.7)	273 (59.6)		767 (58.7)	112 (58.6)	160 (60.4)	
**Menopausal status**															
Postmenopausal	746 (53.8)	206 (54.5)	0,82	746 (53.8)	41 (51.3)	120 (53.3)	45 (61.6)	0,57	714 (54.7)	238 (52.0)	0,32	714 (54.7)	90 (47.1)	147 (55.5)	0,13
Premenopausal	640 (46.2)	172 (45.5)		640 (46.2)	39 (48.8)	105 (46.7)	28 (38.4)		592 (45.3)	220 (48.0)		592 (45.3)	101 (52.9)	118 (44.5)	
**Tumor size**															
≤2	516 (37.3)	123 (32.5)	0,091	516 (37.3)	21 (26.3)	76 (33.8)	26 (35.6)	0,20	463 (35.5)	176 (38.4)	0,26	463 (35.5)	74 (38.7)	102 (38.5)	0,49
>2	869 (62.7)	255 (67.5)		869 (62.7)	59 (73.8)	149 (66.2)	47 (64.4)		842 (64.5)	282 (61.6)		842 (64.5)	117 (61.3)	163 (61.5)	
**Positive lymph nodes**															
0-3	833 (60.1)	224 (59.3)	0,77	833 (60.1)	44 (55.0)	138 (61.3)	42 (57.5)	0,76	766 (58.7)	291 (63.5)	0,066	766 (58.7)	111 (58.1)	178 (67.2)	0,031
≥4	553 (39.9)	154 (40.7)		553 (39.9)	36 (45.0)	87 (38.7)	31 (42.5)		540 (41.3)	167 (36.5)		540 (41.3)	80 (41.9)	87 (32.8)	
**Histological grade**															
I	105 (7.6)	8 (2.1)	<0.001	105 (7.6)	1 (1.3)	7 (3.1)		<0.001	61 (4.7)	52 (11.4)	<0.001	61 (4.7)	22 (11.6)	30 (11.3)	<0.001
II	682 (49.4)	110 (29.2)		682 (49.4)	23 (29.1)	68 (30.2)	19 (26.0)		569 (43.8)	223 (48.8)		569 (43.8)	98 (51.6)	125 (47.2)	
III	593 (43.0)	259 (68.7)		593 (43.0)	55 (69.6)	150 (66.7)	54 (74.0)		670 (51.5)	182 (39.8)		670 (51.5)	70 (36.8)	110 (41.5)	
**Histological type**															
Invasive ductal	1,111 (80.2)	331 (87.6)	<0.001	1,111 (80.2)	70 (87.5)	200 (88.9)	61 (83.6)	0,001	1,082 (82.8)	360 (78.6)	0,001	1,082 (82.8)	147 (77.0)	211 (79.6)	0,008
Invasive lobular	141 (10.2)	16 (4.2)		141 (10.2)	4 (5.0)	9 (4.0)	3 (4.1)		100 (7.7)	57 (12.4)		100 (7.7)	26 (13.6)	31 (11.7)	
Mixed	75 (5.4)	8 (2.1)		75 (5.4)	3 (3.8)	4 (1.8)	1 (1.4)		55 (4.2)	28 (6.1)		55 (4.2)	12 (6.3)	16 (6.0)	
Other	59 (4.3)	23 (6.1)		59 (4.3)	3 (3.8)	12 (5.3)	8 (11.0)		69 (5.3)	13 (2.8)		69 (5.3)	6 (3.1)	7 (2.6)	
**Surgery (binary)**															
MRM	801 (57.8)	208 (55.0)	0,34	801 (57.8)	44 (55.0)	123 (54.7)	41 (56.2)	0,81	744 (57.0)	265 (57.9)	0,74	744 (57.0)	110 (57.6)	155 (58.5)	0,90
Other	585 (42.2)	170 (45.0)		585 (42.2)	36 (45.0)	102 (45.3)	32 (43.8)		562 (43.0)	193 (42.1)		562 (43.0)	81 (42.4)	110 (41.5)	
**Hormonotherapy**															
No	258 (18.7)	145 (38.5)	<0.001	258 (18.7)	36 (45.0)	78 (34.7)	31 (43.1)	<0.001	332 (25.5)	71 (15.5)	<0.001	332 (25.5)	23 (12.0)	47 (17.7)	<0.001
Yes	1,124 (81.3)	232 (61.5)		1,124 (81.3)	44 (55.0)	147 (65.3)	41 (56.9)		969 (74.5)	387 (84.5)		969 (74.5)	168 (88.0)	218 (82.3)	
**Radiotherapy**															
No	341 (25.3)	73 (19.7)	0,026	341 (25.3)	13 (16.7)	47 (21.4)	13 (18.1)	0,12	299 (23.6)	115 (25.7)	0,37	299 (23.6)	45 (24.5)	70 (26.7)	0,56
Yes	1,005 (74.7)	297 (80.3)		1,005 (74.7)	65 (83.3)	173 (78.6)	59 (81.9)		969 (76.4)	333 (74.3)		969 (76.4)	139 (75.5)	192 (73.3)	
**Subtypes, entire cohort**															
Luminal A	539 (38.9)	49 (12.9)	<0.001	539 (38.9)	10 (12.3)	31 (13.8)	8 (11.0)	<0.001	393 (30.1)	195 (42.6)	<0.001	393 (30.1)	100 (52.4)	94 (35.5)	<0.001
Luminal B	369 (26.6)	94 (24.8)		369 (26.6)	19 (23.5)	62 (27.6)	13 (17.8)		327 (25.0)	136 (29.7)		327 (25.0)	45 (23.6)	91 (34.3)	
Luminal HER2	240 (17.3)	78 (20.6)		240 (17.3)	14 (17.3)	49 (21.8)	15 (20.5)		254 (19.4)	64 (14.0)		254 (19.4)	24 (12.6)	40 (15.1)	
HER2-Enriched	88 (6.3)	73 (19.3)		88 (6.3)	20 (24.7)	42 (18.7)	11 (15.1)		131 (10.0)	30 (6.6)		131 (10.0)	11 (5.8)	19 (7.2)	
TNBC	150 (10.8)	85 (22.4)		150 (10.8)	18 (22.2)	41 (18.2)	26 (35.6)		202 (15.5)	33 (7.2)		202 (15.5)	11 (5.8)	21 (7.9)	
**Subtypes, entire cohort, combined**															
Luminal A/B	908 (65.5)	143 (37.7)	<0.001	908 (65.5)	29 (35.8)	93 (41.3)	21 (28.8)	<0.001	720 (55.1)	331 (72.3)	<0.001	720 (55.1)	145 (75.9)	185 (69.8)	<0.001
HER2-positive	328 (23.7)	151 (39.8)		328 (23.7)	34 (42.0)	91 (40.4)	26 (35.6)		385 (29.5)	94 (20.5)		385 (29.5)	35 (18.3)	59 (22.3)	
TNBC	150 (10.8)	85 (22.4)		150 (10.8)	18 (22.2)	41 (18.2)	26 (35.6)		202 (15.5)	33 (7.2)		202 (15.5)	11 (5.8)	21 (7.9)	
**Subtypes concordant***															
Luminal A	467 (46.7)	39 (15.7)	<0.001	467 (46.7)	10 (17.2)	23 (16.2)	6 (12.2)	<0.001	336 (37.3)	170 (49.0)	<0.001	336 (37.3)	85 (60.3)	84 (41.2)	<0.001
Luminal B	314 (31.4)	74 (29.7)		314 (31.4)	16 (27.6)	48 (33.8)	10 (20.4)		269 (29.9)	119 (34.3)		269 (29.9)	38 (27.0)	81 (39.7)	
Luminal-HER2	103 (10.3)	47 (18.9)		103 (10.3)	8 (13.8)	30 (21.1)	9 (18.4)		124 (13.8)	26 (7.5)		124 (13.8)	9 (6.4)	17 (8.3)	
HER2-Enriched	45 (4.5)	46 (18.5)		45 (4.5)	14 (24.1)	24 (16.9)	8 (16.3)		77 (8.5)	14 (4.0)		77 (8.5)	4 (2.8)	10 (4.9)	
TNBC	70 (7.0)	43 (17.3)		70 (7.0)	10 (17.2)	17 (12.0)	16 (32.7)		95 (10.5)	18 (5.2)		95 (10.5)	5 (3.5)	12 (5.9)	
**Subtypes concordant combined**															
Luminal A/B	781 (78.2)	113 (45.4)	<0.001	781 (78.2)	26 (44.8)	71 (50.0)	16 (32.7)	<0.001	605 (67.1)	289 (83.3)	<0.001	605 (67.1)	123 (87.2)	165 (80.9)	<0.001
HER2-positive	148 (14.8)	93 (37.3)		148 (14.8)	22 (37.9)	54 (38.0)	17 (34.7)		201 (22.3)	40 (11.5)		201 (22.3)	13 (9.2)	27 (13.2)	
TNBC	70 (7.0)	43 (17.3)		70 (7.0)	10 (17.2)	17 (12.0)	16 (32.7)		95 (10.5)	18 (5.2)		95 (10.5)	5 (3.5)	12 (5.9)	
**Basal**															
Basal	213 (16.2)	155 (42.5)	<0.001	213 (16.2)	37 (48.1)	85 (39.0)	33 (47.1)	<0.001	301 (24.4)	67 (15.0)	<0.001	301 (24.4)	22 (11.8)	44 (17.1)	<0.001
Non-Basal	1,105 (83.8)	210 (57.5)		1,105 (83.8)	40 (51.9)	133 (61.0)	37 (52.9)		935 (75.6)	380 (85.0)		935 (75.6)	165 (88.2)	214 (82.9)	
**RandomGroup**															
E-CMF	58 (4.2)	28 (7.4)	0,020	58 (4.2)	5 (6.2)	19 (8.4)	4 (5.4)	0,069	64 (4.9)	22 (4.8)	0,29	64 (4.9)	10 (5.2)	12 (4.5)	0,24
E-CMF-Doc	140 (10.1)	42 (11.1)		140 (10.1)	8 (9.9)	31 (13.8)	3 (4.1)		139 (10.6)	43 (9.4)		139 (10.6)	22 (11.5)	21 (7.9)	
E-CMF-T	152 (11.0)	47 (12.4)		152 (11.0)	10 (12.3)	28 (12.4)	9 (12.2)		146 (11.2)	53 (11.6)		146 (11.2)	27 (14.1)	25 (9.4)	
E-T-CMF	834 (60.2)	225 (59.2)		834 (60.2)	51 (63.0)	125 (55.6)	49 (66.2)		769 (58.8)	290 (63.3)		769 (58.8)	110 (57.6)	179 (67.5)	
ET-CMF	202 (14.6)	38 (10.0)		202 (14.6)	7 (8.6)	22 (9.8)	9 (12.2)		190 (14.5)	50 (10.9)		190 (14.5)	22 (11.5)	28 (10.6)	

Notes: MRM: modified radical mastectomy; *: same ER/PgR and HER2 status upon local and central testing; TAD: transactivation domain; DBD: DNA binding domain; TETRA: oligomerization domain.
