# Supplementary material for: TP53 mutations and protein immunopositivity may predict for poor outcome but also for trastuzumab benefit in patients with early breast cancer treated in the adjuvant setting
Source: Oncotarget. 2016 Apr 26;7(22):32731–53. doi: 10.18632/oncotarget.9022 (PMC5078047; doi:10.18632/oncotarget.9022)
Supplement: Supplementary file 3 [file oncotarget-07-32731-s003.pdf]

Table S5: Univariate Cox analysis for clinicopathological parameters in the entire cohort against disease-free survival.

	ENTIRE COHORT					LUMINAL A/B					TNBC					HER2-positive, pre-trastuzumab					HER2-positive, post-trastuzumab				
Parameter	N patients	N events	HR	95% CI	Wald's p	N patients	N events	HR	95% CI	Wald's p	N patients	N events	HR	95% CI	Wald's p	N patients	N events	HR	95% CI	Wald's p	N patients	N events	HR	95% CI	Wald's p
**Age (years)**																									
>50 vs. ≤50	1040 vs. 724	242 vs. 147	1,18	0.96-1.45	0,110	620 vs. 432	132 vs. 86	1,11	0.84-1.45	0,47	136 vs. 99	38 vs. 21	1,38	0.81-2.36	0,23	117 vs. 85	48 vs. 26	1,41	0.87-2.27	0,16	167 vs. 110	24 vs. 14	1,07	0.55-2.07	0,85
**Histological grade**					0,004					0,001					0,049					0,64					0,66
II vs. I	792 vs. 113	171 vs. 11	2,28	1.24-4.20	0,008	559 vs. 98	109 vs. 8	2,39	1.17-4.9	0,017	50 vs. 5	20 vs. 1	1,94	0.26-14.48	0,52	81 vs. 6	30 vs. 1	2,38	0.33-17.48	0,39	103 vs. 4	12 vs. 1	0,49	0.06-3.77	0,49
III vs. I	852 vs. 113	207 vs. 11	2,66	1.45-4.88	0,002	389 vs. 98	101 vs. 8	3,31	1.61-6.79	0,001	180 vs. 5	38 vs. 1	0,99	0.14-7.19	0,99	115 vs. 6	43 vs. 1	2,56	0.35-18.6	0,35	169 vs. 4	25 vs. 1	0,64	0.09-4.69	0,66
**Histological type**					0,042					0,28					0,37					0,60					0,46
Invasive lobular vs. Ductal	157 vs. 1442	34 vs. 316	0,92	0.64-1.31	0,640	133 vs. 817	29 vs. 165	1,01	0.68-1.5	0,97	12 vs. 187	3 vs. 50	0,82	0.25-2.65	0,74	6 vs. 175	2 vs. 64	0,89	0.22-3.62	0,87	6 vs. 265	0 vs. 37	0,47	0.03-8.38	0,60
Mixed vs. Ductal	83 vs. 1442	28 vs. 316	1,49	1.01-2.19	0,044	66 vs. 817	20 vs. 165	1,40	0.88-2.22	0,16	5 vs. 187	2 vs. 50	1,76	0.43-7.25	0,43	9 vs. 175	5 vs. 64	1,69	0.68-4.21	0,26	3 vs. 265	1 vs. 37	3,64	0.68-19.5	0,13
Other vs. Ductal	82 vs. 1442	11 vs. 316	0,57	0.31-1.04	0,065	36 vs. 817	4 vs. 165	0,52	0.19-1.41	0,20	31 vs. 187	4 vs. 50	0,46	0.16-1.26	0,13	12 vs. 175	3 vs. 64	0,66	0.21-2.11	0,48	3 vs. 265	0 vs. 37	1,14	0.07-20.0	0,93
**Subtypes**					0,089																				
Luminal B vs. Luminal A	463 vs. 588	115 vs. 103	1,38	1.06-1.81	0,017	-	-	-	-	-	-	-	-	-	-	-	-	-	-	-	-	-	-	-	-
Luminal HER2 vs. Luminal A	318 vs. 588	78 vs. 103	1,33	0.99-1.79	0,056	-	-	-	-	-	-	-	-	-	-	-	-	-	-	-	-	-	-	-	-
HER2-Enriched vs. Luminal A	161 vs. 588	34 vs. 103	1,25	0.85-1.84	0,260	-	-	-	-	-	-	-	-	-	-	-	-	-	-	-	-	-	-	-	-
TNBC vs. Luminal A	235 vs. 588	59 vs. 103	1,47	1.06-2.02	0,019	-	-	-	-	-	-	-	-	-	-	-	-	-	-	-	-	-	-	-	-
**Hormonotherapy**																									
Yes vs. No	1356 vs. 403	290 vs. 97	0,78	0.62-0.98	0,032	1008 vs. 43	206 vs. 11	0,69	0.37-1.26	0,22	33 vs. 199	8 vs. 50	0,74	0.34-1.59	0,44	152 vs. 49	55 vs. 19	0,80	0.47-1.34	0,39	164 vs. 113	21 vs. 17	0,80	0.42-1.52	0,50
**Menopausal status**																									
Post vs. Premenopausal	952 vs. 812	221 vs. 168	1,15	0.94-1.40	0,180	566 vs. 486	125 vs. 93	1,20	0.92-1.58	0,18	123 vs. 112	32 vs. 27	1,06	0.64-1.78	0,81	107 vs. 95	42 vs. 32	1,22	0.77-1.94	0,39	156 vs. 121	22 vs. 16	0,99	0.52-1.89	0,97
**Positive lymph nodes**																									
≥4 vs. 0-3	707 vs. 1057	249 vs. 140	2,72	2.21-3.35	<0.001	425 vs. 627	142 vs. 76	2,75	2.08-3.64	<0.001	82 vs. 153	33 vs. 26	2,49	1.48-4.17	0,001	113 vs. 89	55 vs. 19	2,73	1.62-4.61	<0.001	88 vs. 189	19 vs. 19	2,32	1.23-4.4	0,010
**Radiotherapy**																									
YES vs. NO	1302 vs. 414	307 vs. 73	1,31	1.01-1.69	0,039	784 vs. 240	179 vs. 36	1,50	1.05-2.15	0,026	175 vs. 55	40 vs. 17	0,72	0.41-1.27	0,25	144 vs. 47	57 vs. 13	1,49	0.82-2.73	0,19	200 vs. 73	31 vs. 7	1,59	0.7-3.62	0,27
**Tumor size**																									
>2 vs. ≤2 cm	1124 vs. 639	295 vs. 93	1,85	1.47-2.34	<0.001	658 vs. 394	165 vs. 53	1,90	1.4-2.6	<0.001	145 vs. 90	43 vs. 16	1,78	1-3.17	0,049	158 vs. 43	64 vs. 9	2,36	1.17-4.75	0,016	165 vs. 112	23 vs. 15	1,01	0.53-1.94	0,98

Notes: Luminal A/B: ER/PgR positive, HER2 negative.
