# Supplementary material for: TP53 mutations and protein immunopositivity may predict for poor outcome but also for trastuzumab benefit in patients with early breast cancer treated in the adjuvant setting
Source: Oncotarget. 2016 Apr 26;7(22):32731–53. doi: 10.18632/oncotarget.9022 (PMC5078047; doi:10.18632/oncotarget.9022)
Supplement: Supplementary file 4 [file oncotarget-07-32731-s004.pdf]

TABLE S11: Description of the 4 HeCOG trials for patients with operable high-risk breast cancer.

Trial	Accrual period	N	n	Treatment schedule	Eligibility criteria	Reference
**HE10/97**<br>Australian New Zealand Clinical Trials Registry ACTRN12611000506998	**1997 - 2000**	**595**	**309**	**E-T-CMF:** Epirubicin 110 mg/m2 q 2 weeks x 3 followed by paclitaxel 250 mg/m2 q 2 weeks x 3 followed by cyclophosphamide 840 mg/m2; methotrexate 57 mg/m2; fluorouracil 840 mg/m2 (CMF) q 2 weeks x 3. GCSF support in all cycles. vs. E-CMF: Epirubicin 110 mg/m2 q 2 weeks x 4 followed by CMF q 2 weeks x 4. GCSF support in all cycles.<br><br>Patients with ER/PgR-positive tumors received tamoxifen 20 mg daily for five years. Premenopausal patients received additional treatment with an LH-RH analog for two years. All patients who underwent partial mastectomy or with tumors >5 cm and/or with ≥4 infiltrated axillary nodes, irrespectively of the type of surgery, were irradiated. Radiation therapy and hormonal therapy were administered after the completion of chemotherapy.	Eligible were women with: histologically confirmed epithelial breast cancer; pathological stage T1-3 N1 M0 or T3 N0 M0 [14]; Eastern Cooperative Oncology Group performance status 0-1; normal cardiac function; and adequate bone marrow, hepatic and renal function.	Fountzilas G, Skarlos D, et al. Postoperative dose-dense sequential chemotherapy with epirubicin, followed by CMF with or without paclitaxel, in patients with high-risk operable breast cancer: a randomized phase III study conducted by the Hellenic Cooperative Oncology Group. Ann Oncol. 2005 Nov;16(11):1762-71.
**HE10/00**<br>Australian New Zealand Clinical Trials Registry ACTRN-12609001036202	**2000 - 2005**	**1.086**	**782**	**E-T-CMF:** As in the HE10/97 trial. **vs. ET-CMF:** Epirubicin 83 mg/m2 + Paclitaxel 187 mg/m2 q 3 weeks x 4 followed by cyclophosphamide 840 mg/m2; methotrexate 57 mg/m2; fluorouracil 840 mg/m2 (CMF) q 2 weeks x 3. GCSF support in all cycles.<br><br>Premenopausal patients received hormonal therapy as in the HE10/97 trial. Postmenopausal patients received tamoxifen 20 mg daily for 2-3 years followed 2-3 years of daily examestane 25 mg. Criteria for irradiation were the same as in the HE10/97 trial.	Eligible were women with: histologically confirmed epithelial breast cancer; pathological stage T1-4 N1-2 M0; Eastern Cooperative Oncology Group performance status 0-1; normal cardiac function and adequate bone marrow, hepatic and renal function.	Gogas H, Dafni U, et al. Postoperative dose-dense sequential versus concomitant administration of epirubicin and paclitaxel in patients with node-positive breast cancer: 5-year results of the Hellenic Cooperative Oncology Group HE 10/00 phase III Trial.<br>Breast Cancer Res Treat. 2012 Apr;132(2):609-19.
**HE10/05**<br>Australian New Zealand Clinical Trials Registry<br>ACTRN-12610000151033	**2005 - 2008**	**990**	**793**	**E-T-CMF:** Epirubicin 110 mg/m2 q 2 weeks x 3 followed by paclitaxel 200 mg/m2 q 2 weeks x 3 followed by cyclophosphamide 840 mg/m2; methotrexate 57 mg/m2; fluorouracil 840 mg/m2 (CMF) q 2 weeks x 3. GCSF support in all cycles.<br><br>**vs. E-CMF-wD:** Epirubicin 110 mg/m2 q 2 weeks x 3 followed by CMF q 2 weeks x 3 followed by weekly Docetaxel 35 mg/m2 x 9<br><br>**vs. E-CMF-wT** Epirubicin 110 mg/m2 q 2 weeks x 3 followed by CMF q 2 weeks x 3 followed by weekly paclitaxel 80 mg/m2 x 9. GCSF support in all cycles in E-T-CMF and during the intensified phase of epirubicin and CMF treatments in E-CMF-wD and E-CMF-wT arms.<br><br>Premenopausal patients received hormonal therapy as in the HE10/97 trial. Postmenopausal patients received anastrazole 1 mg daily for 5 years followed 2-3 years of daily examestane 25 mg. Criteria for irradiation were the same as in the HE10/97 trial.<br><br>Patients with HER2-positive tumors were treated with trastuzumab, initially at a dose of 8 mg/kg as a loading dose, and subsequently 6 mg/kg every three weeks for one year. Initially, HER2-positive tumors were considered those with an immunohistochemistry (IHC) score of 3+ (uniform, intense membrane staining of >10% of invasive tumor cells), a fluorescence in situ hybridization (FISH) result of ≥6 HER2 gene copies, or a FISH ratio (HER2 gene signals to chromosome 17 signals) of >2.0. Following the 2007 publication of the American Society of Clinical Oncology/College of American Pathologists guideline recommendations for HER2 testing in breast cancer, the criteria for characterizing a tumor as HER2-positive	Eligible women were older than 18 years with histologically confirmed node-positive (T1-3 N1 M0) or "intermediate risk" according to the 2005 St. Gallen criteria (node negative patients with at least one of the following features: pT > 2 cm, or histological and/or nuclear grade 2-3, or presence of peritumoral vascular invasion, or HER2 gene overexpression and/or amplification, or age <35 years) adenocarcinoma of the breast. Patients had to have breast-conserving surgery with tumor-free margins or modified radical mastectomy, adequate hematologic, hepatic and renal function, performance status of 0 to 1 of the Eastern Cooperative Oncology Group (ECOG) scale, without evidence of significant cardiac disease (a normal left ventricular ejection fraction [LVEF] demonstrated by a Multiple Gated Acquisition [MUGA] scan or echocardiogram).	Fountzilas G, Dafni U, et al. Dose-dense sequential adjuvant chemotherapy followed, as indicated, by trastuzumab for one year in patients with early breast cancer: first report at 5-year median follow-up of a Hellenic Cooperative Oncology Group randomized phase III trial. BMC Cancer. 2014 Jul 15;14:515.
